# The effects of alternative splicing on miRNA binding sites in bladder cancer

**DOI:** 10.1371/journal.pone.0190708

**Published:** 2018-01-04

**Authors:** Seonggyun Han, Dongwook Kim, Manu Shivakumar, Young-Ji Lee, Tullika Garg, Jason E. Miller, Ju Han Kim, Dokyoon Kim, Younghee Lee

**Affiliations:** 1 Department of Biomedical Informatics, University of Utah School of Medicine, Salt Lake City, Utah, United States of America; 2 Department of Biomedical & Translational Informatics, Geisinger Health System, Danville, Pennsylvania, United States of America; 3 Department of Biomedical Informatics, School of Nursing, University of Pittsburgh, Pittsburgh, Pennsylvania, United States of America; 4 Mowad Urology Department, Geisinger Health System, Danville, Pennsylvania, United States of America; 5 Seoul National University Biomedical Informatics, Seoul, South Korea; Kunming University of Science and Technology, CHINA

## Abstract

Eukaryotic organisms have developed a variety of mechanisms to regulate translation post-transcriptionally, including but not limited to the use of miRNA silencing in many species. One method of post-transcriptional regulation is through miRNAs that bind to the 3′ UTRs to regulate mRNA abundance and influence protein expression. Therefore, the diversity of mRNA 3′ UTRs mediating miRNA binding sites influence miRNA-mediated regulation. Alternative polyadenylation, by shortening mRNA isoforms, increases the diversity of 3′ UTRs; moreover, short mRNA isoforms elude miRNA-medicated repression. Because no current prediction methods for putative miRNA target sites consider whether or not 1) splicing-informed miRNA binding sites and/or 2) the use of 3′ UTRs provide higher resolution or functionality, we sought to identify not only the genome-wide impact of using exons in mRNA 3′ UTRs but also their functional connection to miRNA regulation and clinical outcomes in cancer. With a genome-wide expression of mRNA and miRNA quantified by 395 bladder cancer cases from The Cancer Genome Atlas (TCGA), we 1) demonstrate the diversity of 3′ UTRs affecting miRNA efficiency and 2) identify a set of genes clinically associated with mRNA expression in bladder cancer. Knowledge of 3′ UTR diversity will not only be a useful addition to current miRNA target prediction algorithms but also enhance the clinical utility of mRNA isoforms in the expression of mRNA in cancer. Thus, variability among cancer patient’s variability in molecular signatures based on these exon usage events in 3′ UTR along with miRNAs in bladder cancer may lead to better prognostic/treatment strategies for improved precision medicine.

## Introduction

The 3′ untranslated region (UTR), located at the 3′ end of mRNA, is not translated into protein. However, the 3′ UTR includes important sequences that affect not only the fate of the mRNA, but also regulation by non-coding RNA that influences the total amount of protein [1]. microRNA (miRNA) is a type of non-coding RNA molecule approximately 22 nucleotides in length, which can post transcriptionally regulate gene expression. miRNA primarily binds to its target mRNA at the 3′ UTR of target mRNA of protein-coding genes, which results in repressed translation of mRNA or cleaved mRNA [2–4].

The diversity of mRNA 3′ UTRs in human genes is influenced by alternative polyadenylation (APA) and alternative splicing (AS). In general, mRNA stability is affected by the length of 3′ UTRs because longer mRNA 3′ UTRs provide more regulatory regions, which results in less protein production by degrading its mRNA or attenuating translation [5]. In other words, because of the alternative usage of polyadenylation, shorter mRNA 3′ UTR isoforms lose miRNA binding sites, which initiates not only an evasion of miRNA-mediated inhibition, but also reduces transcriptional capacity [6]. Shorter mRNA isoforms have been identified in the proto-oncogene IGF2BP1/IMP-1 [7]. Moreover, the reduced expression of longer 3′ UTRs is widespread and correlated with proliferation in immune cells [8]. Furthermore, miRNA-mediated repression of mRNA isoforms influences cancer development [9, 10]. For example, the overexpression of the cell cycle gene, Cyclin D1 (*CCND1)*, is very common for many cancers, including bladder cancer, and contributes to cancer development [11–14]. While *CCND1* gene generates two mRNA isoforms [15, 16], the shorter mRNA isoform, which excludes miRNA binding site, results in higher expression of *CCNDI*. Taken together, these results suggest that APA that affects miRNA binding is important for dissecting the etiology of cellular tumorigenesis [17].

AS allows many eukaryotic organisms to generate multiple mRNA isoforms from a single gene through diverse combinations of exons and introns. Exon skipping, intron retention, and alternative 5′ and 3′ splice sites are prevalent events that increase the diversity of mRNA isoforms [18]. Approximately 90% of human genes undergo AS and produce various mRNA isoforms, which results from a different combination of exons [19]. Such diverse exon usage via AS (i.e., exon skipping, intron retention, and alternative 5′ and 3′ splice sites) can occur within 3′ UTR of the mRNA, possibly influencing the ability of miRNA recognition of its target mRNA.

APA can result in shorter mRNA 3′ UTRs, resulting in lower expression of the synthesized protein. Intron retention demonstrates a potential connection between human miRNA targets and mRNA 3′ UTRs, by increasing putative miRNA targets within the 3′ UTRs of human mRNAs [20].

Although APA and intron retention have been known to change putative miRNA target sites, other potential dynamics in 3′ UTRs have not been systematically studied in human mRNA. These potential dynamics cause shorter mRNA 3′ UTR because of 1) splicing events (i.e., exon skipping and alternative 5′ and 3′ splices) and 2) mutually exclusive usage of 3′ UTR region (i.e., no common 3′ UTR region between mRNA isoforms and 3′ UTRs—coding regions in other mRNA isoforms). The objective of this study was to determine the genome-wide impact of different usage of exons in mRNA 3′ UTRs which contain miRNA binding sites, and their clinical relevance. Our hypothesis was that absence of an entire or partial exon in mRNA 3′ UTRs allows mRNA to evade miRNA repression; therefore, miRNA-mediated repression mediates the functional consequence of changed mRNA post-transcription and oncologic outcomes. We tested this hypothesis for muscle invasive bladder cancer using the genome-wide expression of mRNA and miRNA quantified in 395 bladder cancer cases from the Cancer Genome Atlas (TCGA).

## Material and methods

### Data

miRNA expression and RNA-Seq datasets for all cases of bladder urothelial carcinoma were downloaded from the online Genomic Data Commons (GDC) Data Portal (https://gdc-portal.nci.nih.gov/) and Broad Institute GDAC Firehose (http://gdac.broadinstitute.org/) in September 2016. The GDC Data Portal featured 430 cases for RNA-Seq and 437 cases for miRNA. We downloaded 395 cases that matched in both the RNA-Seq and miRNA data sets and clinical information of Stage and Survival (See Methods below). The RNA-seq data for all cases were obtained by Illumina HiSeq and reads featured strand-specific paired-ends. miRNA data was quantified by using a miRNA-seq maturation process. For each bladder cancer case (n = 395), we mapped the RNA-seq reads to the reference genome (i.e., release 75, GRCh37.75) using TopHat v2.1.1 [21]. Additionally, to quantify all mRNA expression levels (i.e., fragment per kilobase per million—FPKM), the mapped bam files from TopHat were assembled using Cufflinks, a tool for comprehensive expression analysis of RNA-seq data [22], with the GTF guide and normalization of the upper quartile-norm options. The miRNA-seq data was downloaded from the Broad Institute GDAC Firehose (analysis version 2016_01_28). In particular, we used the mature miRNA data that was generated from *read per million miRNA mature reads* in level 3 data. All the RPM values from the mature strands were summed and log transformed to generate the miRNA data matrix.

### Compiling miRNA-binding sites from publically available sources

As most existing miRNA target prediction methods are based on the sequence similarity between mature miRNA and the 3′ UTR region of mRNA. These sequence-based miRNA target predictions frequently produce false-positive binding sites due to the lack of exponential validation. Therefore, to obtain comprehensive relations between miRNA and mRNA, we compiled one experimentally validated set with two existing miRNA target prediction databases: 1) miRTarBase, which is based on experimentally validated miRNA targets by reporter assay, western blots, and etc. [23]; 2) TargetScan (Release 7.0), which is based on conserved complementarity between targets of miRNAs and mRNAs [24]; 3) MicroRNA.org, which is based on the miRanda algorithm, and from which only predictions with high confidence scores (alignment score ≥ 120 and binding energy ≤-7.0) were included in this study [25]. Our compilation with data quality control followed three steps: First, we identified experimentally validated pairs between mRNAs and miRNAs using miRTarBase. Second, for the pairs with experimental validations, we obtained mRNA-target sites in 3′ UTR and their genomic coordinates by matching miRNA IDs (i.e., hsa-miR-199) with TargetScan and MicroRNA.org. Third, we then defined 3′ UTR regions of each mRNA based on Ensembl (Release version 75) reference information (http://ftp.ensembl.org/pub/release-75/gtf/homo_sapiens/, September 2016). This compilation yielded 439,404 pairs of miRNAs and their target sites, which is 2.7% of the total predictions (16,228,619 pairs) reported in the MicroRNA.org database. The resultant dataset consists of 1) the genomic coordinates of all miRNA binding sites in 3′ UTRs, 2) the target gene id (i.e., ENSG id), 3) the target mRNA name (i.e., ENST id), and 4) target exons in 3′ UTRs. In our study, *miRNA binding exon* (MBE) refers to any exons including at least one miRNA target site in 3′ UTR of mRNA.

### 3′ UTR miRNA binding site annotations

**Fig 1:**
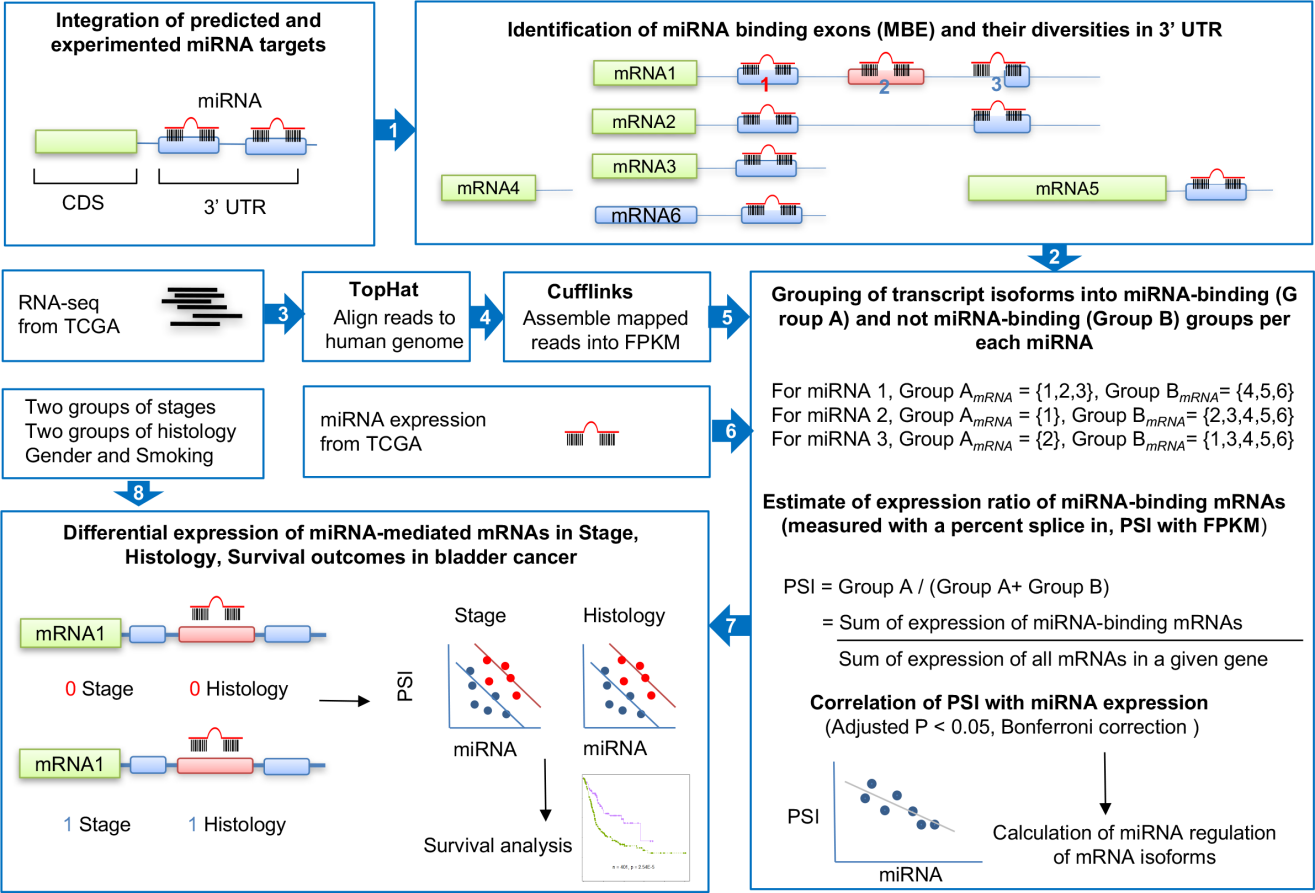
Study design overview. Step 1 involved constructing a comprehensive set of relationships between mRNA and miRNA by compiling three existing miRNA target databases: miTarBase, TargetScan, and miRanda. Step 2 involved searching for the miRNA-binding exons (MBEs) and identifying which transcript isoforms retain or do not retain MBEs. When a transcript isoform loses miRNA binding sites in the 3′ UTR due to one of these events—i.e., 1) exon skipping (miRNA 2 in mRNA2), 2) alternative splice 3′ or 5′ splice sites (miRNA 3 in mRNA1), 3) mutually exclusive 3′ UTR regions (i.e. mRNA3 vs. mRNA5 and mRNA5 vs. mRNA6), defining the case that miRNA binding sites in the genomic regions translated into the two mRNAs do not overlap each other at all, and 4) others in these three cases (i.e., retained introns, non-coding RNA, and alternative polyadenylation)—it was assigned to miRNA-binding Group A; otherwise, it was assigned to Group B. Steps 3, 4, and 5 were to not only identify alternative splicing isoforms and splicing events, but also calculate FPKM as a quantitative expression level using TopHat and Cufflinks. We used level 3 data for miRNA expression in the TCGA. Step 6 integrated comprehensive sets of MBEs status in 3′ UTRs with the expression of miRNA and mRNA and, therefore, estimated the relative expression ratio between Group A (i.e., transcript isoforms repressed by miRNA, defined by MBE-retaining mRNA) and all mRNA expression. This is a normalized measurement of the miRNA-mediated repression ratio to the overall transcript expressions per single gene. The multiplicity was corrected by the Bonferroni method. Step 7 first tested an association of differential expression of miRNA-mediated transcript isoforms in the two-stage and histology groups and then predicted overall survival time in the bladder cancer cases.

We mapped miRNA binding sites in 3′ UTR exons. Overall, there are two types of mRNA isoforms that do not include MBEs in 3′ UTRs: 1) AS-derived and 2) non-AS-derived. The AS-derived absence of MBEs includes exon skipping, intron retention, and alternative 5′ and 3′ splice sites. On the other hand, the non-AS-derived absence of MBEs includes mutually exclusive 3′ UTR between mRNA isoforms, alternative last exon usage, alternative polyadenylation, and non-coding mRNA. For the AS-derived case, we defined which MBEs are constitutively or alternative spliced by comparing all mRNAs in a given gene. If an MBE is skipped in a mRNA isoform, then the isoform is defined as MBE*-free*, in reference to exon skipping or the loss of an entire exon. If part of the MBE is skipped as a result of the alternative 5′ or 3′ splice sites in a mRNA isoform, then the mRNA isoform is defined as *MBE-free* (see **Fig 1, Step 2**). Otherwise, a given mRNA isoform is a MBE-retaining isoform, which is regulated by miRNA.

### Alternative polyadenylation sites in 3′ UTR

We downloaded the APA sites in normal bladder cells from APASdb [26]. APASdb provides tissue-specific APA sites with the supporting read data. We used APA sites with supporting reads >10.

### Define and estimate expression ratio of mRNA isoforms that are repressed by miRNA

We obtained the quantifications of all mRNA expression levels (i.e., FPKM) using Cufflinks. With the expression data, we estimated a relative ratio of MBE-retaining mRNA expression to the summation of the expression of MBE-retaining and MBE-free mRNA isoforms. This relative ratio is presented by percent splice-in (PSI), which is the fraction of affected expression by a single miRNA binding site in a given gene. As such, PSI is a fair estimate of the potential decrease of mRNA expression by miRNA-mediated repression without all possible MBE-free transcript isoforms. For a specific exon *t*, that is an MBE in a 3′ UTR, the PSI value (denoted as a ratio between two groups of MBE-retaining and MBE-deleting mRNA isoforms t), Ψ_t_, was estimated by using the identification of genetic variants affecting alternative splicing (IVAS), a Bioconductor package, which utilizes the following algorithm [27]:

$$f(\Psi t|3\prime UTR)=\frac{\sum_{i=1}^{n}\left(Xit\right)}{\left(\sum_{s=1}^{n}(Xst)+\sum_{i=1}^{n}\left(Xit\right)\right)}$$

Here, *X*_*it*_ and *X*_*st*_ are the expression of MBE-retaining and MBE-free mRNA isoforms *t*, respectively. Therefore, we obtained expressions of the two groups and estimated the expression ratio. Our assumption was that the sum of *X*_*it*_ would be lower than that of *X*_*st*_ by miRNA targets because transcript isoforms in the *X*_*it*_ group are more likely to be repressed by miRNA.

With each pair of miRNA expression and PSI value (i.e., their ratios), we performed a linear regression to determine whether or not mRNA expression is inversely correlated with miRNA expression. The miRNA expression values were the predictor variable and PSI values of the given exon were the outcome variable. Statistically significant associations were determined if *P*_adj_ (i.e., the Bonferroni correction) was less than 0.05 with anti-correlation (*r* < -0.3).

### Association of mRNA expression with stage and histology groups in bladder cancer cases

We used the clinical status information of 395 bladder cancer cases, which have both the RNA-Seq and miRNA data as described above, obtained from the Broad Institute Firehose (analysis_version_2016_01–28). From this clinical data, we classified the 395 cases into subgroups according to the cancer stage, which were based on 1) the progression—from I to IV—of the disease and 2) the histology status, which comprises non-papillary (i.e., 0) or papillary (i.e., 1). Stage II was grouped together (i.e., 130 cases), and stage III and IV were grouped together (i.e., 265 cases). There were 268 cases that exhibited histology 0 status, and 127 cases exhibited histology 1 status. The 395 cases that had both stage and histology information were carried forward. For mRNA isoforms that contained exons with their PSI value anti-correlated with miRNA expressions, we performed a linear regression to identify which genes show differential expression of MBE-retaining mRNAs of a gene having at least two isoforms (each with and without MBE and potentially repressed by miRNA) between the stages and the histology. Age and smoking status were added to the regression model as covariates. The statistical significance between PSI values and clinical groups was defined as p < 0.05 via Bonferroni correction. To validate the p-value from the regression, we performed a permutation test by generating 1,000 sets of random assignments for the stage and histology of the 395 cases. With 1,000 sets of randomized subgroups, we conducted the same linear regression.

### Survival analysis

We divided the 395 cases into two groups according to the PSI values of mRNA (i.e., high and low) for each gene having a statistically significant differential PSI value between the stage groups and the histology groups. We then carried out a Kaplan-Meier survival analysis to assess differences in overall survival between the two groups of cases with high and low PSI values.

## Results

### Genes escaping miRNA-mediated repression because of the diversity of the mRNA 3′ UTR

An overview of our study is depicted in **Fig 1**, and our results are summarized in **S1 Fig**.

**Fig 2:**
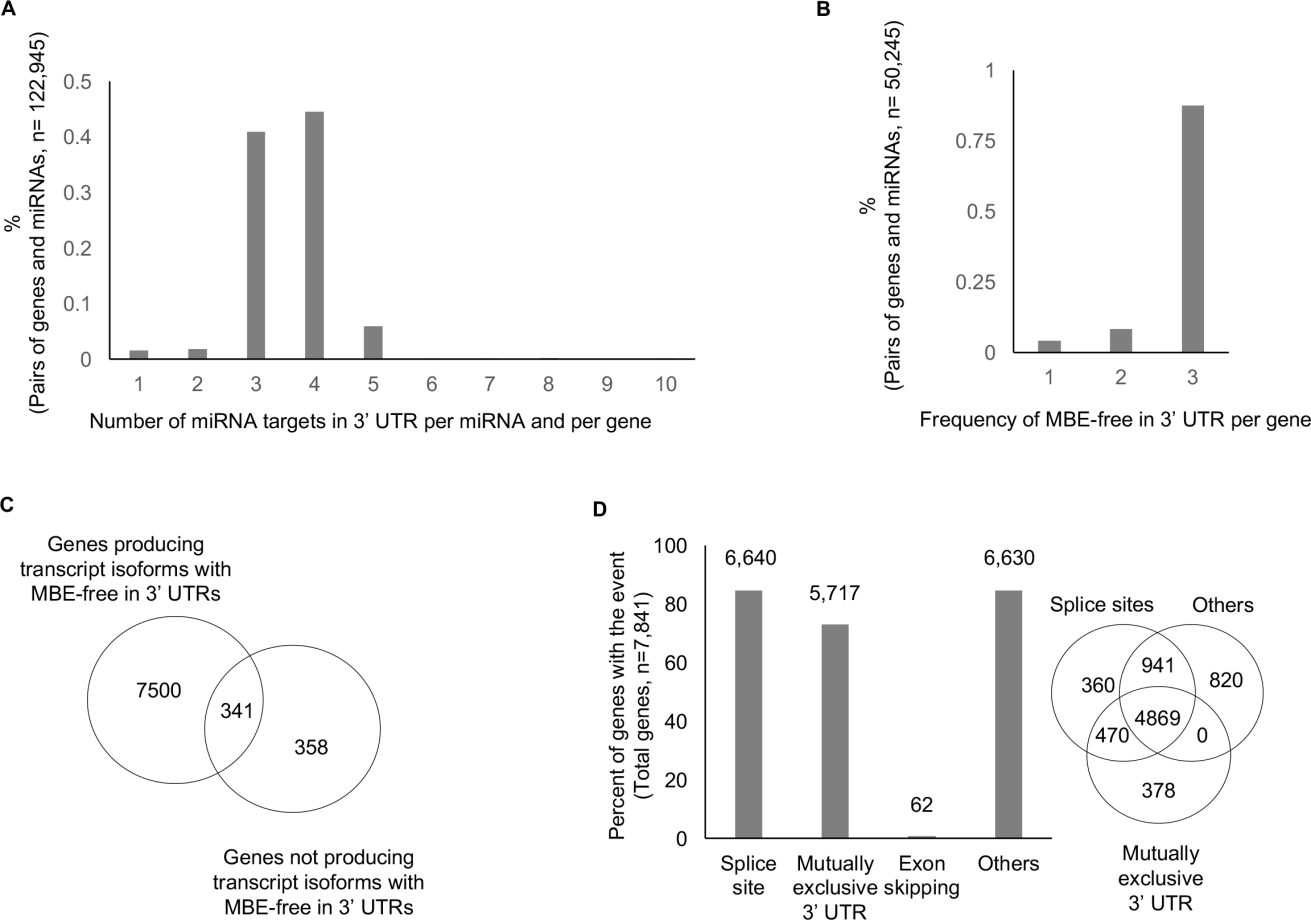
Diversity of mRNA binding exons (MBE) in mRNA 3′ UTR. (A) The figure shows the distribution of miRNA binding sites in 3′ UTRs per miRNA and per gene. Most of the miRNAs bind to three or four different sites in the mRNA 3′ UTRs per gene. (B) For the case of 50,245 pairs that have three miRNA binding sites, most of the pairs (86%) are translated into mRNA without all three miRNA binding sites. (C) The Venn diagram shows the number of genes generating a transcript isoform that can potentially escape from miRNA-mediated repression per single miRNA. (D) As described in Step 2 of Fig 1, transcript isoforms are diverse in their 3′ UTRs, where miRNA binds. The Venn diagram shows the frequencies of MBE-free transcript isoforms in four cases: 1) exon skipping, 2) alternative 5′ and 3′ splice sites, 3) mutually exclusive 3′ UTR regions, and 4) others.

A comprehensive dataset of miRNAs and their target sites was compiled by integrating three repositories of miRNA targets: one experimentally validated miRNA target and two predicted miRNA targets. We obtained the experimentally validated unique pairs of miRNA and target which comprised 1,410 miRNAs and 9,793 genes. Among these 9,793 genes, 9,142 genes had at least two mRNA isoforms, and only these genes were included for further analysis. A summary of our results is depicted in **S1 Fig**. Among the 439,404 pairs of miRNAs and their binding sites, we defined MBEs in 3′ UTRs (see **Materials and Methods**). For the 9,142 genes that generated more than two mRNA isoforms, we identified one mRNA per gene that had multiple miRNA target sites in the 3′ UTR. In addition, 40.9% and 44.5% of genes had three and four miRNA target sites in the 3’ UTR per a single gene and per single miRNA, respectively (see **Fig 2A**). In other words, most of each miRNA have three or four binding sites in 3’ UTR per target gene. Interestingly, among 50,245 pairs that had three miRNA binding sites, 86% lost all three miRNA binding sites, which suggests that at least one mRNA (i.e., miRNA-binding exon [MBE]-free—see Materials and Methods for definition) completely eluded miRNA-mediated repression (see **Fig 2B**). In addition, 86% (i.e., 7,841) of the genes produced at least one such mRNA isoform that is MBE-free (i.e., 313,091 unique pairs of miRNA and their target sites), while a mere 8% (i.e., 699 genes) generated only mRNA isoforms that retain MBE (see **Fig 2C**). Furthermore, we not only examined the diverse features of MBE-free that occurred in 3′ UTRs of every transcript isoform per miRNA per single gene, but also further classified the feature of losing miRNA binding sites in 3′ UTRs into four categories (see **Fig 1**, Step 2): 1) alternative 3′ or 5′ splice sites (i.e., miRNA 3 in mRNA1), 2) mutually exclusive 3′ UTR regions (i.e., miRNA case in mRNA3 vs. mRNA5 and mRNA5 vs. mRNA6), 3) exon skipping (i.e., miRNA 2 in mRNA2), and 4) others (i.e., retained introns, non-coding RNA, and alternative polyadenylation). **Fig 2D** shows the distribution of each feature that results in MBE-free. As the AS-related event, alternative 5′ or 3′ splice sites were the major features that resulted in MBE-free. Moreover, mutually exclusive 3′ UTR regions was a frequent feature that also may contribute to change-final mRNA expression.

### Diversity of mRNA 3′ UTRs conferring multiple transcript isoforms may affect miRNA-mediated repression of mRNAs

We obtained 214,293 unique mRNA isoforms using Cufflinks. There were 395 cases of miRNA expression that matched to cases with RNA-seq data. Only miRNAs that had an expression value in more than 50% of cases (>200 cases) were included in our analysis. As a result, we obtained 548 miRNAs, which corresponded to 166,551 pairs (i.e., 548 miRNAs and 7,607 genes) among 313,091 pairs in 7,841 genes, which generate transcript isoforms that are MBE-free 3′ UTRs (see the right panel of **S1 Fig**, right). Next, using linear regression to identify a statistically significant correlation of miRNA expression with mRNA expression (i.e., PSI) for each of the 166,551 pairs across 548 miRNAs and 7,607 genes in 395 bladder cancer cases, we identified 2,324 statistically significant pairs, which comprised 182 miRNAs and 619 genes (adjusted P_adj_ < 0.05 via Bonferroni correction). Among them, 569 unique pairs (i.e., 78 miRNAs and 155 genes) were inversely correlated (i.e., *r* < -0.3, adjusted p < 0.05, see **S1 Table**, the bottom panel of **S1 Fig**). However, when the miRNAs were tested for correlation with gene level expression, 1,979 pairs (i.e., 299 miRNAs and 1,158 genes) were statistically significant, and 148 pairs (i.e., 47 miRNAs and 103 genes) exhibited anti-correlations. Interestingly, only eight genes were identified in both calculations for mRNA and gene level expression, indicating concordant impacts of miRNA-mediated repression on both levels. In other words, the summation of all mRNAs expressed from a gene is likely to be similar to the given gene’s expression level, which is the level of miRNA-mediated regulation. Such concordance between mRNA and gene-level expression affected by miRNA can be explained by the cases we observed. First, the intrinsic expression level of each transcript isoform differs. For example, when transcript isoforms targeted by a miRNA are predominantly expressed over isoforms not so targeted, the miRNA-targeted mRNAs are major contributors to the total gene expression level. Second, mRNAs have different numbers and locations of miRNA binding sites. For example, when a mRNA has only one miRNA binding site, the mRNA expression level repressed by the given miRNA can be similar to the gene expression level repressed by the miRNA. **S2 Fig.** shows one example, *COLEC12*, out of eight genes that have two transcript isoforms. The mRNA expression level of transcript ENST00000400256 was inversely correlated with expression of hsa-miR-148b-3p, indicating that the miRNA might repress the mRNA. Since the other mRNA isoform, ENST00000582147, is a non-coding RNA with an expression level of almost zero, miRNA-mediated repression showed concordant impacts at the both mRNA and gene levels (**S2D Fig**).

### Links between alternative splicing and alternative polyadenylation

As stated in the introduction, APA is one factor that increases 3′ UTR diversity—a factor well-studied in miRNA regulation. APA in transcript isoforms is known to be linked to alternative splicing [28]. We estimated how many cases among 569 unique pairs were coupled with APA using a database of potential APA sites (**See Methods**). When the APA is located upstream of the alternative splicing events of each pair, these splicing events can be diminished due to the early cleavage site. Among 569 pairs, 27.5% (157 pairs) were predicted to have APA sites upstream of the splicing events, suggesting that splicing events in these 157 pairs are linked to APA. Although our study has limitations such as examining which transcript isoforms differ in 3′UTRs by an APA and whether or not APA is AS-dependent, it benefits from our classification of all possible dynamics in 3′ UTRs; moreover, we tested purely miRNA-binding transcript isoforms for their associations with the given miRNA.

### Differential expressions of MBE-retaining transcript isoforms between stage and histology subgroups in bladder cancer

American Joint Committee on Cancer (AJCC) cancer stage is a standardized way to assess the level of 1) tumor invasion, 2) spread to lymph nodes, and 3) metastasis to distant sites. Cancer stage is often determined through a histology report which will then be used to determine a prognosis. Therefore, by developing more precise measurements for defining stage can potentially inform and improve treatment. Since 3’UTR isoforms were shown to regulate gene expression with respect to bladder cancer, we hypothesized that the 3’ UTR isoforms in bladder cancer samples could potentially differ between cancer stage and histology subgroups, thus providing a new means to assess the state of cancer.

As described in Materials and Methods, we grouped 395 bladder cancer cases into two AJCC stage groups and two histology groups. For 155 genes, which exhibit statistically significant reverse-correlation between the expression of miRNA and mRNA expression, we examined whether or not these mRNA isoforms are differentially expressed between each of the two stage and histology groups. From a regression test, a total of 7 and 37 genes, were identified (see **S1 Table**, bottom and right panel of **S1 Fig**), which were differentially expressed between stage and histology groups (adjusted p < 0.05 via Bonferroni correction), respectively. Seven genes were differentially expressed in both of stage and histology groups.

### Functional enrichment analysis on identified genes

**Fig 3:**
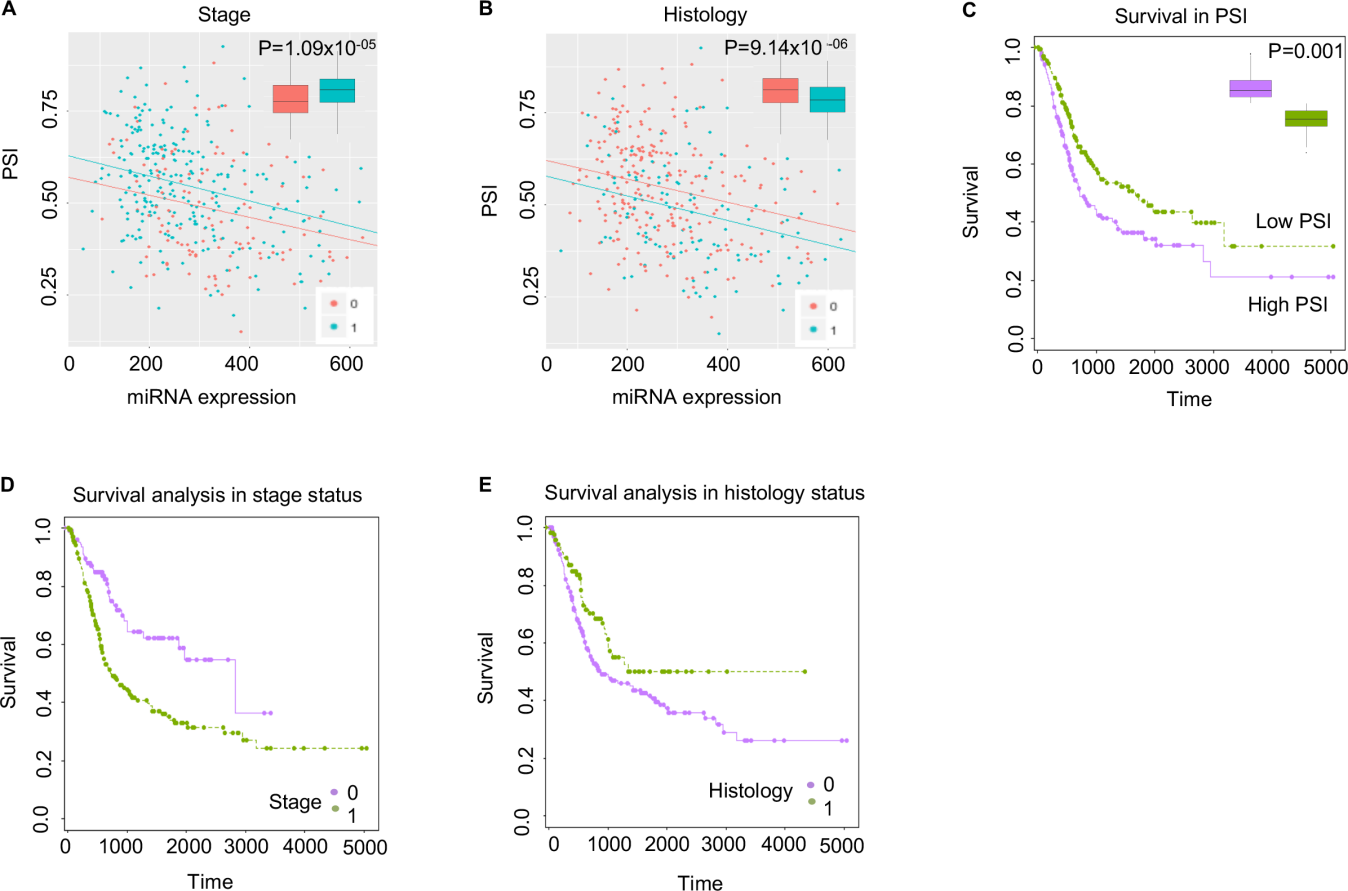
Case study of the *VEGFA* gene, which shows statistically significant differential expression of miRNA-mediated transcript isoforms between stage status, histology status, and survival outcomes. For plots A and B, the x-axes represent miRNA expression, and the y-axes represent the relative ratio of miRNA-mediated transcript isoforms expression to overall transcript isoforms expression per a single miRNA (i.e., hsa-miR-361-5p). The p-value was corrected with the Bonferroni method (A) Red dots and blue dots represent stage 0 and stage 1 of bladder cancer, respectively. (B) Red dots and blue dots represent histology 0 and histology 1 of bladder cancer, respectively. (C) In the two groups of bladder cancer cases that expressed high PSI and low PSI, the later showed better survival outcomes, compared to the latter. As expected, more severe stage group 0 (i.e., purple plot in (D) and histology group 1 (i.e., green plot in E) exhibited a shorter expected survival time.

We performed a pathway over-representation analysis using ConsensusPathDB (CPDB) to identify enriched pathways, given identified genes from the analyses [29]. For 7 genes associated with stage, we observed an enriched KEGG pathway (i.e., miRNAs in cancer) (FDR *q* < 0.05), which suggests that these 7 genes are regulated by previously identified cancer-related miRNAs; moreover, this regulation induces subsequent, malignant effects on cell differentiation, proliferation, and apoptosis that lead to tumor growth and progress. An over representation analysis also demonstrated that 37 genes associated with histology were over-represented in nine pathways (FDR *q* < 0.05) (**S2 Table**). Furthermore, 16 genes associated with survival were enriched in eight pathways, which included photodynamic therapy-induced HIF-1 survival signaling and HIF-1-alpha transcription factor network pathways (**S3 Table**). Hypoxia-inducible factor (HIF)-1 is a transcription factor that regulates genes involved in cancer development and contributes to anaerobic metabolism and angiogenesis [30, 31]. Notably, HIF-1 is a well-known transcriptional factor associated with bladder cancer [32] and other cancers [33], and Nadaoka et al. assert that HIF-1 alpha polymorphism is a prognostic factor in bladder carcinoma [34]. Taken together, these observations suggest that HIF-1 may play an important role in variability, based on the different usage of exons in mRNA 3'UTRs and their functional connection to miRNA regulation efficiency. Therefore, HIFs not only mediate resistance to radio- and chemotherapy, but also are associated with poor prognosis in bladder cancer patients. In addition to the over-representation analysis, we also performed a network analysis of the interaction- and pathway-centric analysis of lists of genes, known as induced network modules analysis [35]. Given 16 genes associated with survival, these seed genes were to interconnect those genes through different types of interactions (e.g., physical, biochemical, and regulatory) (**S3 Fig**). In particular, *VEGFA*, which was associated with stage, histology, and survival, was connected to multiple genes associated with HIF-1 as gene regulatory interactions (**S3 Fig**, see **Fig 3**and below for detail). A previous study supports the evidence of an activation of vascular endothelial growth factor gene transcription by hypoxia-inducible factor 1[36].

### Clinical interpretation

For the 7 and 37 genes that 1) generated mRNA isoforms that lose miRNA target exons and 2) exhibited differential expression in cancer stage and histology, respectively, we examined whether or not these genes inform bladder cancer prognosis for a given patient. Through Kaplan-Meier survival analysis (see Materials and Methods), 5 and 16 genes, respectively, exhibited a statistically significant association with stage and histology (bottom and right panel of **S1 Fig**); meanwhile, five genes exhibited a statistically significant association with both of stage and histology: *CYBRD1*, *H2AFY*, *PCSK7*, *VEGFA*, and *ZFPM2*.

#### VEGFA: Statistical significance for three clinical features

The hsa-miR-361-5p miRNA potentially binds to the 3′ UTR (i.e., chromosome 6, 43,753,919–43,753,940) of mRNA transcribed from the Vascular Endothelial Growth Factor A (*VEGFA*) gene. As shown in **Fig 3**, the PSI value (i.e., the relative expression ratio between mRNA isoforms with and without a miRNA target exon) is likely to be highly expressed in stage group 1 and histology group 0. Moreover, of the two subgroups (i.e., low and high PSI expression), we found that, through K-means clustering, the subgroup with a higher PSI ratio comprised more severe cases. The *VEGFA* gene is part of the platelet-derived growth factor (PDGF) / vascular endothelial growth factor (VEGF) family, which is associated with angiogenesis in cancer. In fact, in an examination of the association between VEGF-A expression in bladder cancer tissues and clinical outcomes, Fauconnet et al. suggest that transcript levels are higher among bladder cancer stages T2 to T4 [37].

#### BACE1: Statistical significance in stage and histology only

**Fig 4:**
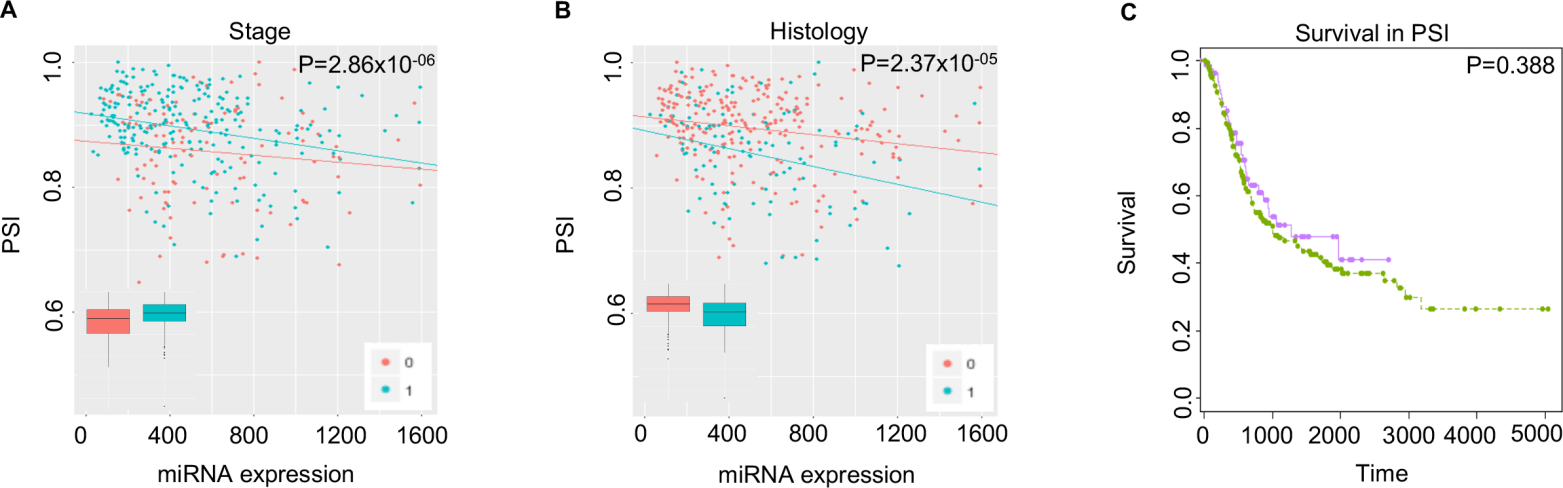
Case study of the *BACE1* gene, which shows statistically significant differential expression of miRNA-mediated transcript isoforms between stage status and histology status—but not survival outcome. For plots A and B, the x-axes represent miRNA expression, and the y-axes represent the relative ratio of miRNA-mediated transcript isoforms expression to overall transcript isoforms expression per single miRNA (i.e., hsa-miR-17-5p). The p-value was corrected with the Bonferroni method. (A) Red dots and blue dots represent stage 0 and stage 1 of bladder cancer, respectively. (B) Red dots and blue dots represent histology 0 and histology 1 of bladder cancer, respectively. (C) In the two groups of bladder cancer cases that expressed high PSI and low PSI, we observed no differences in survival outcome.

Hsa-miR-17-5p miRNA potentially targets the chr11:117160243–117160223 chromosome in the 3′ UTR of the Beta-Secretase 1 (*BACE1*) gene. As shown in **Fig 4**, the PSI level was high for stage group 1 and histology group 0. However, we observed no statistically significant differential survival outcomes between the two subgroups based on low and high PSI values. *BACE1* is a gene that encodes the Beta-secretase 1 (BACE1) enzyme, which has important roles in forming myelin sheaths in peripheral nerves [38]. *BACE1* not only holds clinical relevance in the pathogenesis of Alzheimer’s disease, but also has been a molecular target for drug development [39]. Little is known about the association between *BACE1* and cancer; however, because bladder cancer exhibits the highest median age (i.e., 73 years) at diagnosis of all cancer types, this gene may have an association with disease development in aging populations.

## Discussion

Bladder cancer is the fifth most common malignancy among American men, with 74,000 new cases diagnosed in 2015 [40]. Approximately 25% of bladder cancers are muscle-invasive at diagnosis (i.e., tumor stage T2 or greater), which confers a worse prognosis. The standard treatment for muscle-invasive bladder cancer is platinum-based neoadjuvant chemotherapy followed by radical cystectomy and urinary diversion, which is a major extirpative surgical procedure to remove the bladder and reconstruct the urinary tract [7] with high complication rates [41]. The overall 5-year survival for a muscle-invasive disease is 50%, even with radical treatment [42]. Moreover, few changes in mortality rates or advancements in disease management this disease have been reported over the past 30 years. Clearly, better understanding of the molecular basis of the disease offers the possibility of changing the treatment paradigm to offer personalized, molecular drug target-based approaches based on an individual’s tumor biology, stage, and tumor morphology.

Genes in eukaryote cells undergo post-transcriptional regulation as part of their translation into proteins, and one of the main forms of this regulation is coordinated by miRNA. In the process of this regulation, 3′ UTRs serve as a regulatory sequence onto which miRNA binds and, therefore, adjust the stability of mRNA. Up to 90% of human genes generate multiple mRNA isoforms, and mRNA isoforms can differ in their 3′ UTRs. Our examination of mRNA 3′ UTRs revealed the different features of 3′ UTRs that allow transcript isoforms to elude miRNA-mediated regulation. As a result, our study provides comprehensive knowledge on the diversity of 3′ UTRs, which can function as a useful addition to current miRNA target prediction algorithms [18]. The relative ratio of transcript isoform expression *and* signature differ between cancer and normal tissues; moreover, the ratio characterizes tumor phenotypes and progression—but not gene expression [43–45]. These results also suggest that information about transcript isoforms is necessary for understanding miRNA mediated gene regulatory mechanisms and advancing precision medicine in cancer.

In our study, we explored two sources of omics data (i.e., RNA-seq and miRNA) for bladder cancer from TCGA. In the coming iteration of our research, we will extend our examination to PanCancer in TCGA to further investigate this comprehensive feature of diversity in 3′ UTRs across human cancers. Furthermore, this work helps elucidate the architecture of cancer specific transcript isoform signatures and their regulation by miRNAs through the diversity of 3′ UTRs. In summary, the cancer patient’s characterizing alternative splicing events along with miRNA binding sites in bladder cancer may lead to better prognostic/treatment strategies.

## Supporting information

S1 Fig A summary of results produced in each step described in Fig 1. (PDF)

S2 Fig Concordant impacts of hsa-miR-148b-3p on mRNA and gene-level expression in *COLEC12*. (PDF)

S3 Fig Induced network modules analysis of the associated survival of 16 genes. Nodes with black labels are seed genes; nodes with purple labels are intermediate nodes.
(PDF)

S1 Table Seventy-eight miRNAs and 155 genes that are inversely correlated between mRNA and miRNA expression. (PDF)

S2 Table Pathway over-representation analysis of 37 genes associated with histology. (PDF)

S3 Table Pathway over-representation analysis of 16 genes associated with survival. (PDF)
