# Supplementary figures and images for: The effects of alternative splicing on miRNA binding sites in bladder cancer
Source: PLoS One. 2018 Jan 4;13(1):e0190708. doi: 10.1371/journal.pone.0190708 (PMC5754136; doi:10.1371/journal.pone.0190708)

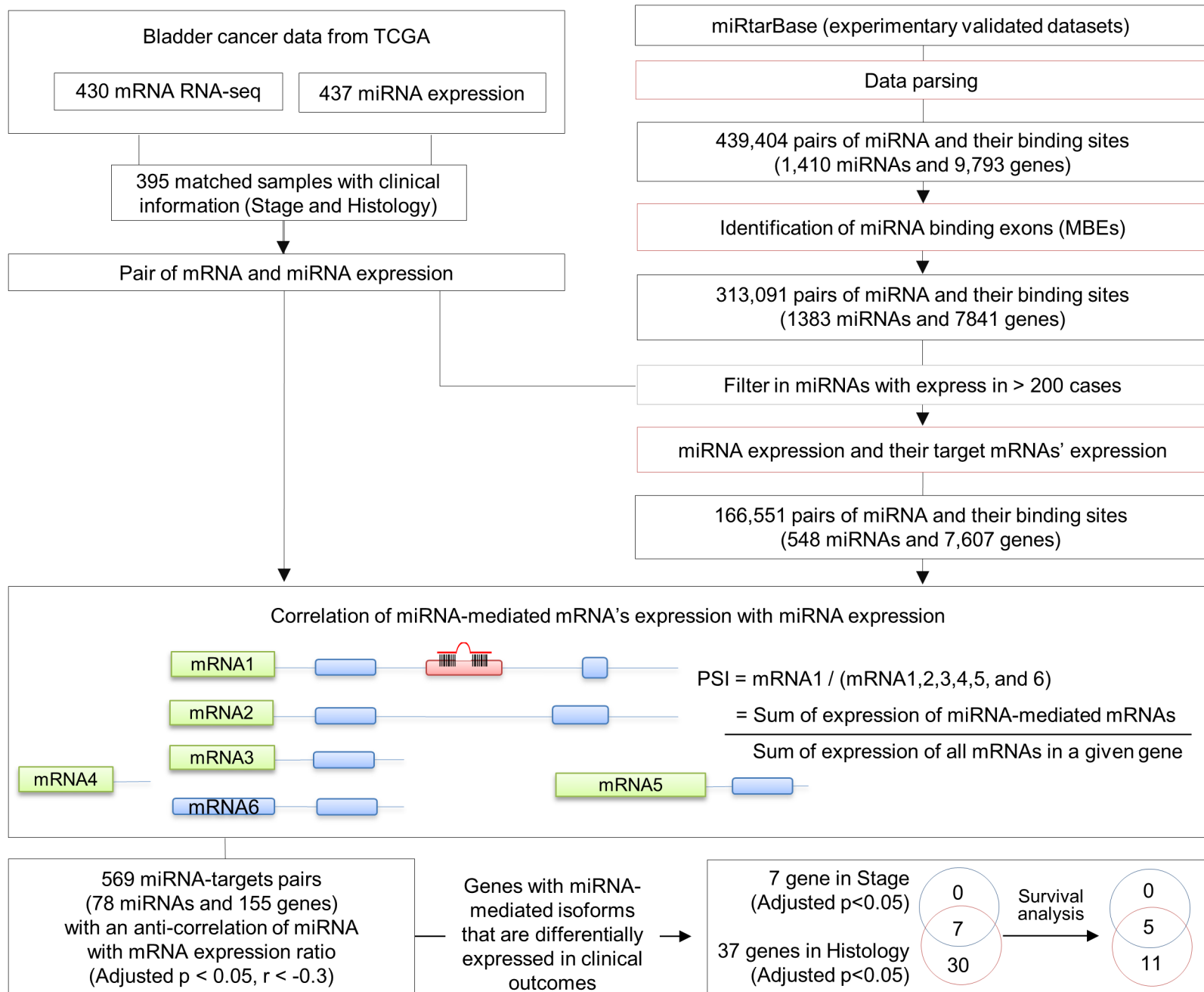

**S1 Fig. A summary of results produced in each step described in Fig 1.**

Supplement: S1 Fig — (PDF) [file pone.0190708.s001.pdf]

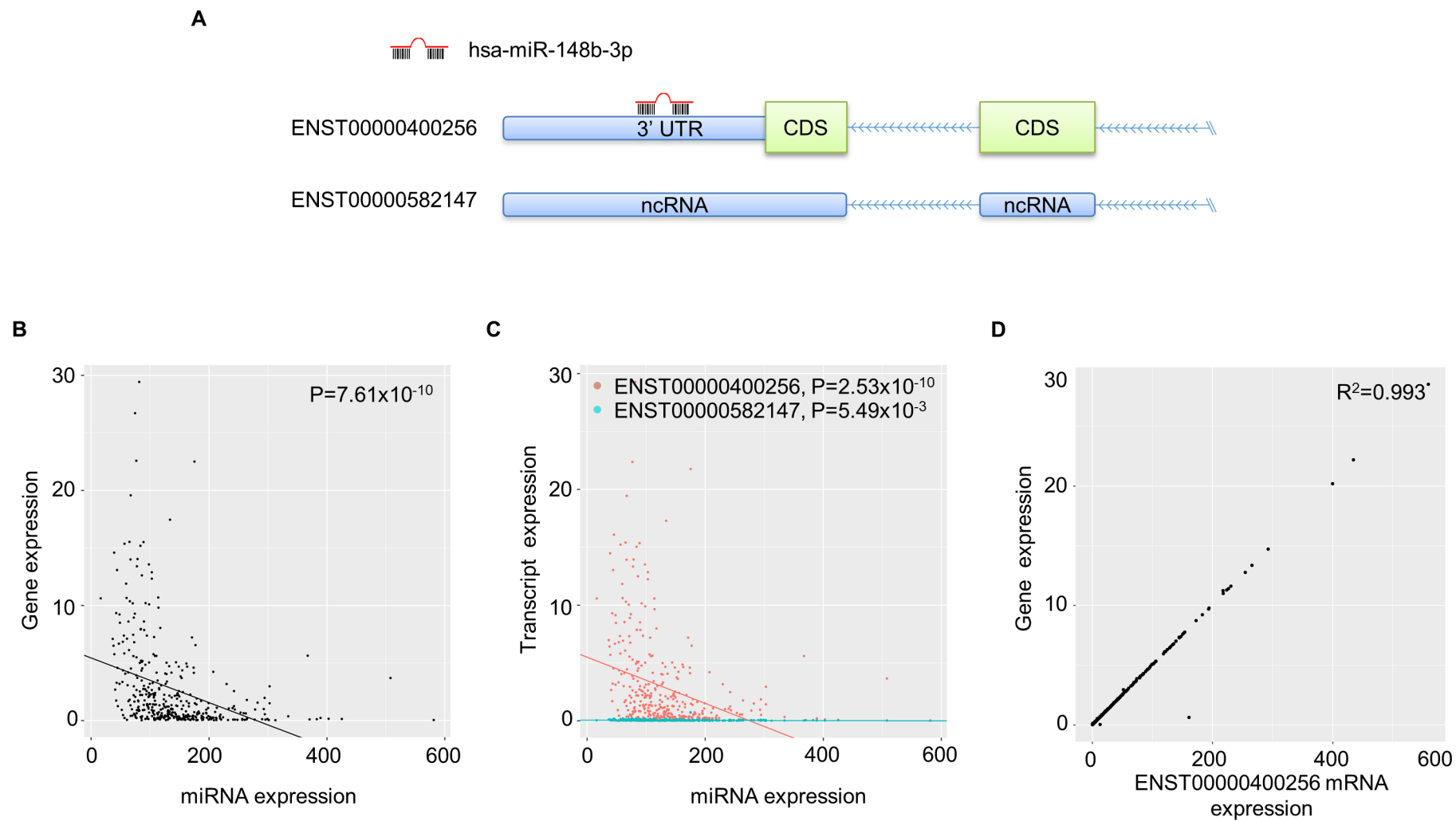

**S2 Fig. Concordant impacts of hsa-miR-148b-3p on mRNA and gene-level expression in COLEC12.**

Supplement: S2 Fig — (PDF) [file pone.0190708.s002.pdf]
