# Supplementary material for: The effects of alternative splicing on miRNA binding sites in bladder cancer
Source: PLoS One. 2018 Jan 4;13(1):e0190708. doi: 10.1371/journal.pone.0190708 (PMC5754136; doi:10.1371/journal.pone.0190708)
Supplement: S3 Fig — Nodes with black labels are seed genes; nodes with purple labels are intermediate nodes. (PDF) [file pone.0190708.s003.pdf]

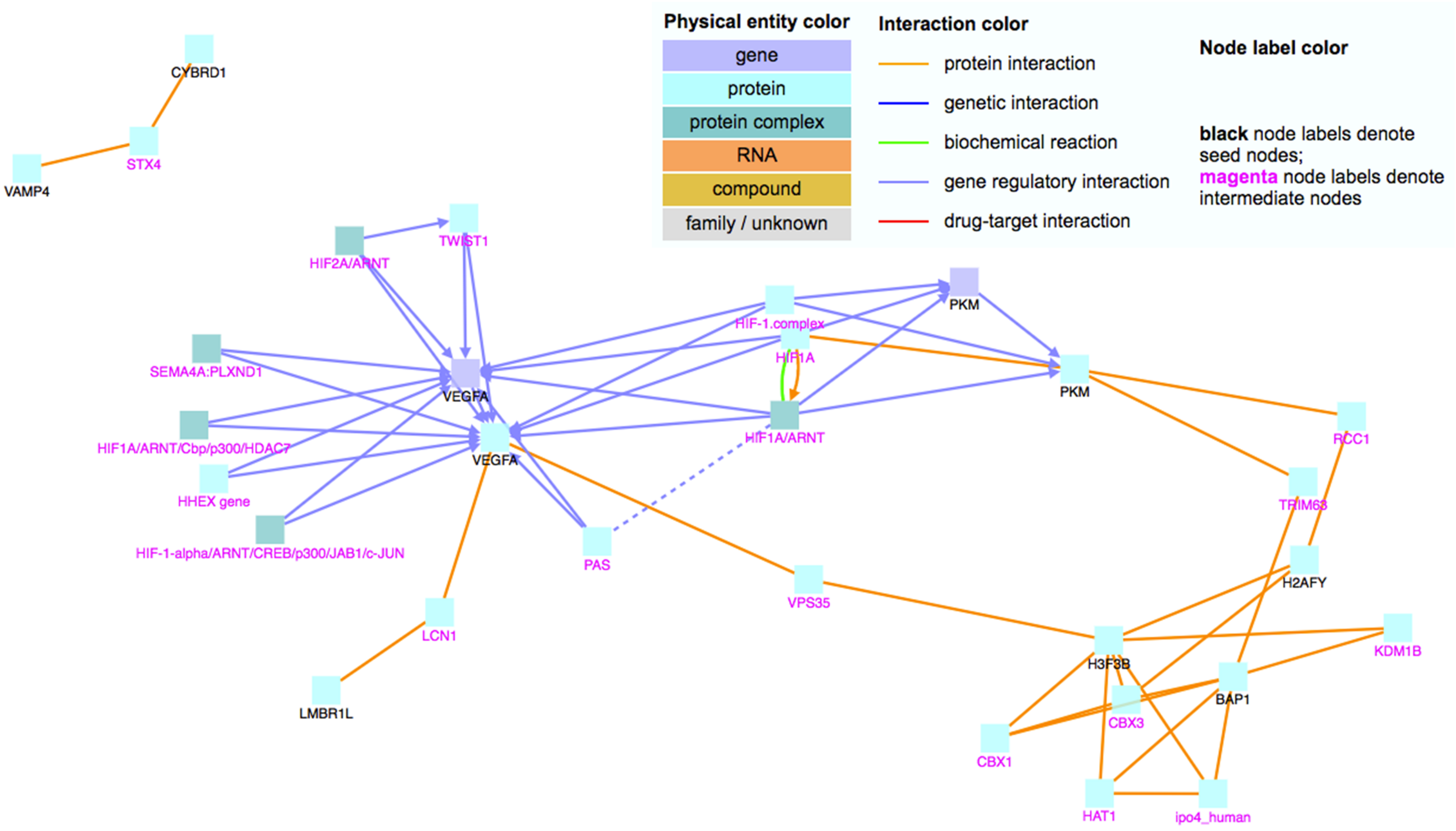

S3 Fig. Induced network modules analysis of the associated survival of 16 genes. Nodes with black labels are seed genes ; nodes with purple labels are intermediate nodes.
