# Supplementary material for: The effects of alternative splicing on miRNA binding sites in bladder cancer
Source: PLoS One. 2018 Jan 4;13(1):e0190708. doi: 10.1371/journal.pone.0190708 (PMC5754136; doi:10.1371/journal.pone.0190708)
Supplement: S1 Table — (PDF) [file pone.0190708.s004.pdf]

Table S1. Seventy-eight miRNAs and 155 genes that are inversely correlated between mRNA and miRNA expression.

Gene symbol	miRNA ID	Target site in 3' UTR	P-value	Correlation
EPB41	hsa-miR-30e-5p	1:29445335-29445358	3.73E-14	-0.358
EPB41	hsa-miR-30e-5p	1:29445459-29445479	3.73E-14	-0.358
EPB41	hsa-miR-30c-5p	1:29445459-29445479	1.38E-10	-0.306
EPB41	hsa-miR-30c-5p	1:29445337-29445358	1.38E-10	-0.306
EPB41	hsa-miR-30e-5p	1:29445243-29445263	3.73E-14	-0.358
S100PBP	hsa-miR-30e-5p	1:33322318-33322339	7.45E-14	-0.354
S100PBP	hsa-miR-30c-5p	1:33322319-33322339	1.32E-13	-0.351
S100PBP	hsa-miR-30c-5p	1:33323446-33323469	4.29E-13	-0.344
S100PBP	hsa-miR-30e-5p	1:33323452-33323469	1.65E-13	-0.349
S100PBP	hsa-miR-30c-5p	1:33324268-33324294	4.29E-13	-0.344
S100PBP	hsa-miR-30e-5p	1:33324282-33324303	1.65E-13	-0.349
SH3GLB1	hsa-miR-21-3p	1:87211679-87211701	1.46E-10	-0.306
SH3GLB1	hsa-miR-29a-5p	1:87209437-87209458	1.03E-10	-0.309
SH3GLB1	hsa-miR-21-3p	1:87210127-87210154	1.46E-10	-0.306
SH3GLB1	hsa-miR-21-3p	1:87210680-87210700	1.46E-10	-0.306
SH3GLB1	hsa-miR-29a-5p	1:87211155-87211176	1.03E-10	-0.309
SH3GLB1	hsa-miR-29a-5p	1:87211192-87211214	1.03E-10	-0.309
SDHC	hsa-miR-320a	1:161332514-161332536	1.11E-13	-0.352
SDHC	hsa-miR-320a	1:161332443-161332466	1.11E-13	-0.352
RCOR3	hsa-miR-200c-3p	1:211486390-211486411	1.71E-13	-0.349
RCOR3	hsa-miR-200c-3p	1:211486565-211486586	1.71E-13	-0.349
RCOR3	hsa-miR-200c-3p	1:211486458-211486483	1.71E-13	-0.349
KCTD3	hsa-miR-30c-5p	1:215794637-215794660	4.75E-11	-0.314
KCTD3	hsa-miR-30c-5p	1:215794430-215794452	4.75E-11	-0.314
KCTD3	hsa-miR-30c-5p	1:215794402-215794424	4.75E-11	-0.314
CAPN2	hsa-miR-101-3p	1:223963052-223963075	8.77E-11	-0.310
CAPN2	hsa-miR-101-3p	1:223963489-223963512	8.77E-11	-0.310
CAPN2	hsa-miR-101-3p	1:223963352-223963374	8.77E-11	-0.310
SRM	hsa-miR-423-5p	1:11114700-11114674	1.37E-13	-0.351
SRM	hsa-miR-423-5p	1:11114763-11114741	1.88E-13	-0.349
SRM	hsa-miR-320a	1:11114735-11114709	1.07E-12	-0.338
SRM	hsa-miR-320a	1:11114823-11114803	9.45E-13	-0.339
SRM	hsa-miR-423-5p	1:11114873-11114852	1.88E-13	-0.349
SRM	hsa-miR-320a	1:11114704-11114686	1.07E-12	-0.338
SFPQ	hsa-miR-141-3p	1:35648913-35648892	1.02E-10	-0.309
C1orf123	hsa-miR-30e-5p	1:53679888-53679864	6.01E-22	-0.446
C1orf123	hsa-miR-30e-5p	1:53679969-53679945	6.01E-22	-0.446
C1orf123	hsa-miR-30e-5p	1:53680032-53680009	6.01E-22	-0.446
TGFBR3	hsa-miR-27a-3p	1:92146323-92146297	4.27E-12	-0.330
SLC16A1	hsa-miR-29c-3p	1:113456449-113456425	1.60E-17	-0.399
SLC16A1	hsa-miR-29c-3p	1:113456170-113456147	1.60E-17	-0.399
SLC16A1	hsa-miR-29c-3p	1:113455473-113455452	1.60E-17	-0.399
SLC16A1	hsa-miR-29c-3p	1:113456503-113456479	1.60E-17	-0.399

CSDE1	hsa-miR-15b-5p	1:115259852-115259827	1.66E-11	-0.321
NOTCH2	hsa-miR-29b-5p	1:120456828-120456805	8.29E-11	-0.312
NOTCH2	hsa-miR-29b-5p	1:120457011-120456987	8.29E-11	-0.312
NOTCH2	hsa-miR-29b-5p	1:120455597-120455574	8.29E-11	-0.312
CDC42SE1	hsa-miR-29c-3p	1:151024524-151024503	2.81E-14	-0.360
CDC42SE1	hsa-miR-29c-3p	1:151025335-151025313	2.81E-14	-0.360
ADAMTS4	hsa-miR-143-3p	1:161159924-161159902	1.45E-13	-0.350
ADAMTS4	hsa-miR-143-3p	1:161160227-161160203	1.45E-13	-0.350
ADAMTS4	hsa-miR-143-3p	1:161160107-161160086	1.45E-13	-0.350
VAMP4	hsa-miR-223-5p	1:171671052-171671031	6.76E-10	-0.303
VAMP4	hsa-miR-223-5p	1:171670144-171670116	5.17E-10	-0.305
MRPS14	hsa-miR-27a-3p	1:174983098-174983079	5.68E-16	-0.381
MRPS14	hsa-miR-27a-3p	1:174982662-174982644	5.68E-16	-0.381
FAM129A	hsa-miR-106a-5p	1:184763760-184763741	2.22E-10	-0.303
FAM129A	hsa-miR-106a-5p	1:184761356-184761334	2.22E-10	-0.303
FAM129A	hsa-miR-20b-5p	1:184763767-184763741	1.05E-10	-0.308
FAM129A	hsa-miR-20b-5p	1:184761356-184761334	1.05E-10	-0.308
FAM129A	hsa-miR-17-5p	1:184763760-184763741	2.13E-10	-0.303
FAM129A	hsa-miR-106a-5p	1:184763789-184763769	2.22E-10	-0.303
FAM129A	hsa-miR-92a-3p	1:184763363-184763343	4.95E-11	-0.314
FAM129A	hsa-miR-92a-3p	1:184762666-184762649	4.95E-11	-0.314
FAM129A	hsa-miR-20b-5p	1:184763789-184763769	1.05E-10	-0.308
FAM129A	hsa-miR-17-5p	1:184761356-184761334	2.13E-10	-0.303
FAM129A	hsa-miR-92a-3p	1:184763779-184763756	4.95E-11	-0.314
FAM129A	hsa-miR-93-5p	1:184761356-184761334	4.00E-15	-0.371
FAM129A	hsa-miR-92a-3p	1:184764105-184764082	4.95E-11	-0.314
FAM129A	hsa-miR-17-5p	1:184763789-184763769	2.13E-10	-0.303
FAM129A	hsa-miR-93-5p	1:184763760-184763741	4.00E-15	-0.371
FAM129A	hsa-miR-93-5p	1:184760903-184760881	4.00E-15	-0.371
SNX17	hsa-let-7g-5p	2:27599681-27599702	1.66E-16	-0.388
SNX17	hsa-let-7g-5p	2:27599723-27599740	1.66E-16	-0.388
CLIP4	hsa-miR-15b-5p	2:29405489-29405517	1.20E-11	-0.323
CLIP4	hsa-miR-15b-5p	2:29405832-29405857	1.20E-11	-0.323
CLIP4	hsa-miR-15b-5p	2:29405456-29405479	1.20E-11	-0.323
PRKCE	hsa-miR-21-5p	2:46412628-46412653	1.55E-13	-0.350
PRKCE	hsa-miR-21-5p	2:46414518-46414537	1.55E-13	-0.350
PRKCE	hsa-miR-21-5p	2:46413112-46413137	1.55E-13	-0.350
UGGT1	hsa-miR-21-5p	2:128951157-128951179	5.02E-11	-0.313
UGGT1	hsa-miR-21-5p	2:128949573-128949602	5.02E-11	-0.313
CYBRD1	hsa-miR-92a-3p	2:172412677-172412698	3.36E-17	-0.396
CYBRD1	hsa-miR-92a-3p	2:172413296-172413322	3.36E-17	-0.396
CYBRD1	hsa-miR-92a-3p	2:172412457-172412479	3.36E-17	-0.396
XRCC5	hsa-miR-29b-5p	2:217070725-217070751	3.18E-10	-0.303
XRCC5	hsa-miR-29b-5p	2:217070413-217070440	3.18E-10	-0.303
XRCC5	hsa-miR-29b-5p	2:217070281-217070304	3.18E-10	-0.303
GPC1	hsa-miR-149-3p	2:241406705-241406725	2.84E-13	-0.383
GPC1	hsa-miR-149-5p	2:241407258-241407279	3.03E-10	-0.301

GPC1	hsa-miR-149-5p	2:241405900-241405929	3.03E-10	-0.301
GPC1	hsa-miR-149-5p	2:241405800-241405823	3.03E-10	-0.301
GPC1	hsa-miR-149-3p	2:241406926-241406948	2.84E-13	-0.383
GPC1	hsa-miR-149-3p	2:241405969-241405990	2.84E-13	-0.383
DNMT3A	hsa-miR-143-3p	2:25456067-25456044	6.55E-14	-0.355
DNMT3A	hsa-miR-143-3p	2:25456826-25456806	6.55E-14	-0.355
DNMT3A	hsa-miR-143-3p	2:25456967-25456950	6.55E-14	-0.355
SLC8A1	hsa-miR-1-3p	2:40340448-40340427	1.22E-18	-0.412
SLC8A1	hsa-miR-1-3p	2:40341112-40341091	1.22E-18	-0.412
SLC8A1	hsa-miR-1-3p	2:40340549-40340525	1.22E-18	-0.412
MCFD2	hsa-miR-191-5p	2:47130496-47130478	3.39E-11	-0.316
MCFD2	hsa-miR-191-5p	2:47130942-47130923	3.39E-11	-0.316
MCFD2	hsa-miR-191-5p	2:47130874-47130849	3.39E-11	-0.316
SMEK2	hsa-miR-106b-5p	2:55776147-55776126	1.23E-10	-0.307
SMEK2	hsa-miR-106b-5p	2:55777026-55777006	1.23E-10	-0.307
SMEK2	hsa-miR-106b-5p	2:55776382-55776362	1.23E-10	-0.307
SMEK2	hsa-miR-106b-5p	2:55776244-55776224	1.23E-10	-0.307
MRPS5	hsa-miR-320a	2:95752975-95752953	4.84E-11	-0.314
SEMA4C	hsa-let-7g-5p	2:97526168-97526145	2.48E-13	-0.347
SEMA4C	hsa-miR-125a-5p	2:97525794-97525768	4.32E-11	-0.314
SEMA4C	hsa-let-7g-5p	2:97526355-97526331	2.48E-13	-0.347
SEMA4C	hsa-miR-125a-5p	2:97526309-97526285	4.32E-11	-0.314
SEMA4C	hsa-let-7g-5p	2:97526313-97526289	2.48E-13	-0.347
SEMA4C	hsa-miR-125a-5p	2:97525833-97525809	4.32E-11	-0.314
SEMA4C	hsa-let-7g-5p	2:97526355-97526333	2.48E-13	-0.347
SEMA4C	hsa-let-7g-5p	2:97525916-97525898	2.48E-13	-0.347
GTF3C3	hsa-miR-29b-5p	2:197629184-197629163	3.45E-11	-0.318
GTF3C3	hsa-miR-29b-5p	2:197629261-197629237	3.45E-11	-0.318
ACY1	hsa-miR-96-5p	3:52023104-52023123	1.57E-10	-0.306
ACY1	hsa-miR-96-5p	3:52023096-52023120	1.57E-10	-0.306
FNDC3B	hsa-miR-155-5p	3:172115927-172115951	5.86E-11	-0.312
BAP1	hsa-miR-429	3:52436232-52436214	5.13E-14	-0.356
BAP1	hsa-miR-141-3p	3:52436232-52436214	2.54E-10	-0.302
BAP1	hsa-miR-200c-3p	3:52436234-52436214	4.27E-12	-0.330
BAP1	hsa-miR-200a-3p	3:52435545-52435525	5.73E-12	-0.328
BAP1	hsa-miR-200a-3p	3:52435596-52435574	5.73E-12	-0.328
BAP1	hsa-miR-429	3:52435460-52435435	2.95E-11	-0.317
BAP1	hsa-miR-429	3:52435546-52435525	2.95E-11	-0.317
BAP1	hsa-miR-200b-3p	3:52436232-52436214	1.38E-18	-0.411
BAP1	hsa-miR-200b-3p	3:52435459-52435435	2.36E-15	-0.374
BAP1	hsa-miR-200b-3p	3:52435545-52435525	2.36E-15	-0.374
BAP1	hsa-miR-200a-3p	3:52435421-52435398	5.73E-12	-0.328
NT5DC2	hsa-miR-423-3p	3:52558480-52558458	2.62E-11	-0.318
NT5DC2	hsa-miR-423-3p	3:52558453-52558429	2.62E-11	-0.318
KLHL6	hsa-miR-10a-5p	3:183208354-183208330	1.50E-14	-0.363
KLHL6	hsa-miR-10a-5p	3:183209698-183209674	1.50E-14	-0.363
KLHL6	hsa-miR-10a-5p	3:183207003-183206977	1.50E-14	-0.363

ACAP2	hsa-miR-129-5p	3:194999390-194999370	7.39E-13	-0.347
ACAP2	hsa-miR-129-5p	3:194998072-194998046	7.39E-13	-0.347
ACAP2	hsa-miR-129-5p	3:194997575-194997553	7.39E-13	-0.347
PCGF3	hsa-let-7i-5p	4:759869-759891	1.88E-10	-0.304
FBXL5	hsa-miR-130b-3p	4:15607307-15607286	1.60E-11	-0.321
FBXL5	hsa-miR-17-5p	4:15606657-15606637	1.59E-15	-0.376
FBXL5	hsa-miR-17-5p	4:15607305-15607284	1.59E-15	-0.376
FBXL5	hsa-miR-93-5p	4:15607305-15607284	2.81E-14	-0.360
FBXL5	hsa-miR-93-5p	4:15606937-15606915	2.81E-14	-0.360
LCORL	hsa-miR-21-5p	4:17884549-17884525	1.01E-11	-0.324
LCORL	hsa-miR-21-5p	4:17884691-17884670	1.01E-11	-0.324
LCORL	hsa-miR-21-5p	4:17883188-17883167	1.01E-11	-0.324
ARAP2	hsa-miR-101-3p	4:36069222-36069198	1.67E-11	-0.321
ARAP2	hsa-miR-101-3p	4:36069517-36069493	1.67E-11	-0.321
ARAP2	hsa-miR-101-3p	4:36068144-36068122	1.67E-11	-0.321
ARAP2	hsa-miR-101-3p	4:36068618-36068597	1.67E-11	-0.321
GNPDA2	hsa-miR-106b-5p	4:44705072-44705054	1.74E-21	-0.442
GNPDA2	hsa-miR-106b-5p	4:44704487-44704461	4.81E-20	-0.427
GNPDA2	hsa-miR-106b-5p	4:44704248-44704226	4.81E-20	-0.427
FAM13A	hsa-miR-2355-5p	4:89648836-89648816	6.14E-11	-0.312
FAM13A	hsa-miR-2355-5p	4:89648088-89648068	2.19E-10	-0.303
FAM13A	hsa-miR-2355-5p	4:89647229-89647207	2.19E-10	-0.303
RNF150	hsa-miR-148b-3p	4:141787875-141787853	3.15E-12	-0.331
RNF150	hsa-miR-148b-3p	4:141788505-141788486	3.15E-12	-0.331
RNF150	hsa-miR-148b-3p	4:141788992-141788971	3.15E-12	-0.331
YTHDC2	hsa-miR-30c-5p	5:112929409-112929431	3.35E-10	-0.300
YTHDC2	hsa-miR-30c-5p	5:112929264-112929290	3.35E-10	-0.300
LYRM7	hsa-miR-21-5p	5:130538995-130539018	1.30E-10	-0.307
LYRM7	hsa-miR-21-5p	5:130540654-130540685	1.30E-10	-0.307
LYRM7	hsa-miR-21-5p	5:130538114-130538136	1.30E-10	-0.307
KDM3B	hsa-miR-101-3p	5:137772268-137772296	2.91E-10	-0.301
KDM3B	hsa-miR-101-3p	5:137772529-137772556	2.91E-10	-0.301
MTMR12	hsa-miR-1-3p	5:32228952-32228931	9.99E-15	-0.366
MTMR12	hsa-miR-1-3p	5:32227852-32227829	9.99E-15	-0.366
MTMR12	hsa-miR-1-3p	5:32227711-32227686	9.99E-15	-0.366
ANKRA2	hsa-miR-30e-5p	5:72848218-72848198	3.95E-13	-0.344
ANKRA2	hsa-miR-30e-5p	5:72848606-72848585	3.95E-13	-0.344
ANKRA2	hsa-miR-30e-5p	5:72848580-72848552	3.95E-13	-0.344
H2AFY	hsa-miR-148b-3p	5:134670644-134670623	4.35E-11	-0.314
H2AFY	hsa-miR-148b-3p	5:134670499-134670476	3.33E-11	-0.316
H2AFY	hsa-miR-148b-3p	5:134670312-134670288	3.33E-11	-0.316
H2AFY	hsa-miR-148b-3p	5:134670468-134670447	3.33E-11	-0.316
NR3C1	hsa-miR-155-5p	5:142657855-142657828	2.53E-25	-0.478
CAMK2A	hsa-miR-149-3p	5:149601884-149601863	3.46E-10	-0.334
CAMK2A	hsa-miR-149-3p	5:149600531-149600509	3.46E-10	-0.334
CAMK2A	hsa-miR-149-3p	5:149599941-149599920	3.46E-10	-0.334
BOD1	hsa-miR-29b-5p	5:173035276-173035252	3.92E-12	-0.332

PDLIM7	hsa-miR-1-3p	5:176917469-176917448	5.19E-26	-0.484
PDLIM7	hsa-miR-1-3p	5:176917309-176917290	5.19E-26	-0.484
PDLIM7	hsa-miR-1-3p	5:176917275-176917254	5.19E-26	-0.484
BTN3A1	hsa-miR-106b-5p	6:26414110-26414129	5.52E-15	-0.369
BTN3A1	hsa-miR-106b-5p	6:26414317-26414338	3.90E-14	-0.358
BTN3A1	hsa-miR-106b-5p	6:26415073-26415093	3.90E-14	-0.358
BTN3A3	hsa-miR-106b-5p	6:26452990-26453013	2.81E-10	-0.302
PTK7	hsa-miR-324-5p	6:43128957-43128995	9.58E-11	-0.309
PTK7	hsa-miR-324-5p	6:43128653-43128675	2.20E-10	-0.303
PTK7	hsa-miR-324-5p	6:43128783-43128805	9.58E-11	-0.309
PTK7	hsa-miR-324-5p	6:43128621-43128641	2.20E-10	-0.303
VEGFA	hsa-miR-101-3p	6:43754048-43754069	2.09E-10	-0.304
VEGFA	hsa-miR-101-3p	6:43753528-43753549	2.09E-10	-0.304
VEGFA	hsa-miR-200c-3p	6:43753598-43753620	1.52E-18	-0.411
VEGFA	hsa-miR-200c-3p	6:43754105-43754131	1.52E-18	-0.411
VEGFA	hsa-miR-93-5p	6:43754190-43754212	2.76E-11	-0.317
VEGFA	hsa-miR-200c-3p	6:43753745-43753772	1.52E-18	-0.411
VEGFA	hsa-miR-101-3p	6:43753372-43753393	2.09E-10	-0.304
VEGFA	hsa-miR-200b-3p	6:43753599-43753620	2.16E-25	-0.478
VEGFA	hsa-miR-200b-3p	6:43752956-43752974	2.16E-25	-0.478
VEGFA	hsa-miR-200b-3p	6:43754106-43754131	2.16E-25	-0.478
VEGFA	hsa-miR-15a-5p	6:43752575-43752598	5.40E-11	-0.313
VEGFA	hsa-miR-106b-5p	6:43753095-43753114	1.84E-11	-0.320
VEGFA	hsa-miR-106b-5p	6:43754192-43754212	1.84E-11	-0.320
VEGFA	hsa-miR-93-5p	6:43752478-43752499	1.29E-12	-0.337
VEGFA	hsa-miR-141-5p	6:43753153-43753173	7.48E-11	-0.311
VEGFA	hsa-miR-93-5p	6:43753088-43753114	2.76E-11	-0.317
VEGFA	hsa-miR-361-5p	6:43752363-43752384	1.01E-10	-0.309
VEGFA	hsa-miR-141-5p	6:43753256-43753277	7.48E-11	-0.311
VEGFA	hsa-miR-106b-5p	6:43752481-43752499	7.57E-13	-0.340
VEGFA	hsa-miR-361-5p	6:43753919-43753940	1.52E-10	-0.306
VEGFA	hsa-miR-141-5p	6:43752597-43752619	6.05E-11	-0.312
GCLC	hsa-miR-30c-5p	6:53362324-53362303	2.44E-17	-0.397
GCLC	hsa-miR-30e-5p	6:53362527-53362505	1.69E-27	-0.496
GCLC	hsa-miR-30e-5p	6:53362330-53362309	1.69E-27	-0.496
GCLC	hsa-miR-30c-5p	6:53363220-53363199	2.44E-17	-0.397
GCLC	hsa-miR-30d-5p	6:53362321-53362303	1.61E-10	-0.305
GCLC	hsa-miR-30c-5p	6:53362408-53362384	2.44E-17	-0.397
GCLC	hsa-miR-30e-5p	6:53363221-53363199	1.69E-27	-0.496
GCLC	hsa-miR-30d-5p	6:53363221-53363199	1.61E-10	-0.305
GCLC	hsa-miR-30d-5p	6:53362526-53362505	1.61E-10	-0.305
MAP3K7	hsa-miR-15a-5p	6:91226218-91226203	2.85E-10	-0.301
MAP3K7	hsa-miR-15a-5p	6:91226108-91226085	2.85E-10	-0.301
MAP3K7	hsa-miR-15a-5p	6:91225903-91225888	2.85E-10	-0.301
MAP3K7	hsa-miR-15a-5p	6:91225965-91225938	2.85E-10	-0.301
SOD2	hsa-miR-222-5p	6:160103372-160103348	2.13E-10	-0.303
SOD2	hsa-miR-222-5p	6:160103454-160103432	2.13E-10	-0.303

SOD2	hsa-miR-222-5p	6:160103079-160103056	6.23E-12	-0.327
SOD2	hsa-miR-130a-5p	6:160103001-160102974	5.21E-11	-0.339
SOD2	hsa-miR-130a-5p	6:160103338-160103314	5.70E-10	-0.321
SOD2	hsa-miR-130a-5p	6:160102788-160102768	5.21E-11	-0.339
WIPI2	hsa-miR-15a-5p	7:5270763-5270784	2.02E-12	-0.334
WIPI2	hsa-miR-15a-5p	7:5270626-5270650	2.62E-12	-0.333
WIPI2	hsa-miR-15a-5p	7:5270583-5270607	2.62E-12	-0.333
RAC1	hsa-miR-320a	7:6442730-6442749	8.44E-13	-0.340
RAC1	hsa-miR-320a	7:6443257-6443278	8.44E-13	-0.340
RAC1	hsa-miR-320a	7:6442231-6442257	8.44E-13	-0.340
RAC1	hsa-miR-320a	7:6442080-6442099	7.67E-12	-0.326
AVL9	hsa-miR-146b-5p	7:32623729-32623751	5.79E-12	-0.328
SUMF2	hsa-miR-148b-3p	7:56147327-56147349	1.27E-11	-0.323
CROT	hsa-miR-17-5p	7:87028527-87028549	1.31E-10	-0.307
CROT	hsa-miR-17-5p	7:87028332-87028356	1.31E-10	-0.307
CROT	hsa-miR-33a-5p	7:87028232-87028259	9.87E-11	-0.310
CROT	hsa-miR-33a-5p	7:87028315-87028334	9.87E-11	-0.310
CROT	hsa-miR-33a-5p	7:87028163-87028183	9.87E-11	-0.310
CROT	hsa-miR-17-5p	7:87027965-87027986	1.31E-10	-0.307
COL1A2	hsa-miR-29c-3p	7:94060322-94060342	9.02E-11	-0.309
COL1A2	hsa-miR-29c-3p	7:94059835-94059856	9.02E-11	-0.309
SNX13	hsa-miR-21-3p	7:17833665-17833649	2.89E-14	-0.360
SNX13	hsa-miR-21-3p	7:17833664-17833643	2.89E-14	-0.360
GTF2IRD2	hsa-miR-27a-3p	7:74210589-74210569	5.44E-12	-0.328
GTF2IRD2	hsa-miR-27a-3p	7:74210646-74210627	5.44E-12	-0.328
GTF2IRD2	hsa-miR-27a-3p	7:74210539-74210521	5.44E-12	-0.328
C7orf43	hsa-miR-30e-5p	7:99752178-99752157	7.17E-12	-0.326
C7orf43	hsa-miR-30e-5p	7:99752510-99752491	1.63E-15	-0.376
C7orf43	hsa-miR-30e-5p	7:99752347-99752326	7.17E-12	-0.326
ADAM9	hsa-miR-30e-5p	8:38961553-38961571	1.63E-10	-0.305
ADAM9	hsa-miR-30e-5p	8:38962149-38962173	1.63E-10	-0.305
IKBKB	hsa-miR-151a-3p	8:42189745-42189768	1.13E-17	-0.401
IKBKB	hsa-miR-200c-3p	8:42189899-42189925	3.34E-23	-0.458
IKBKB	hsa-miR-200c-3p	8:42188499-42188520	3.78E-20	-0.428
IKBKB	hsa-miR-200c-3p	8:42188886-42188907	2.21E-23	-0.460
IKBKB	hsa-miR-200c-3p	8:42189384-42189405	5.51E-13	-0.342
IKBKB	hsa-miR-151a-3p	8:42188498-42188518	5.55E-12	-0.328
IKBKB	hsa-miR-151a-3p	8:42189365-42189385	3.00E-10	-0.301
ZFPM2	hsa-miR-429	8:106815965-106815985	1.14E-10	-0.308
ZFPM2	hsa-miR-200c-3p	8:106816500-106816524	1.27E-29	-0.513
ZFPM2	hsa-miR-429	8:106816501-106816524	2.19E-10	-0.303
ZFPM2	hsa-miR-200b-3p	8:106815776-106815803	3.48E-14	-0.359
ZFPM2	hsa-miR-200c-3p	8:106815775-106815803	1.49E-28	-0.505
ZFPM2	hsa-miR-200c-3p	8:106815962-106815985	4.08E-30	-0.517
ZFPM2	hsa-miR-141-3p	8:106815966-106815985	6.38E-24	-0.465
ZFPM2	hsa-miR-200b-3p	8:106815966-106815985	1.38E-14	-0.364
ZFPM2	hsa-miR-200b-3p	8:106816501-106816524	3.34E-14	-0.359

ZFPM2	hsa-miR-200a-3p	8:106816501-106816524	1.72E-15	-0.375
ZFPM2	hsa-miR-141-3p	8:106816501-106816524	1.10E-23	-0.463
ZFPM2	hsa-miR-141-3p	8:106815783-106815803	7.50E-21	-0.435
ZFPM2	hsa-miR-200a-3p	8:106815776-106815803	1.65E-14	-0.363
ZFPM2	hsa-miR-200a-3p	8:106815966-106815985	6.74E-16	-0.380
ZFPM2	hsa-miR-429	8:106815783-106815803	3.24E-10	-0.301
ZFAND1	hsa-miR-125a-5p	8:82614919-82614897	4.14E-13	-0.344
ZFAND1	hsa-miR-125a-5p	8:82614343-82614323	9.00E-13	-0.339
ZFAND1	hsa-miR-125a-5p	8:82614031-82614008	9.00E-13	-0.339
DERL1	hsa-miR-27a-3p	8:124026222-124026202	1.70E-10	-0.305
DERL1	hsa-miR-27a-3p	8:124027498-124027479	6.47E-13	-0.341
EHMT1	hsa-miR-30e-5p	9:140729418-140729441	5.26E-11	-0.313
EHMT1	hsa-miR-30e-5p	9:140730499-140730522	5.26E-11	-0.313
EHMT1	hsa-miR-30e-5p	9:140729979-140730000	5.26E-11	-0.313
EHMT1	hsa-miR-30e-5p	9:140730480-140730497	5.26E-11	-0.313
STOML2	hsa-miR-320a	9:35099967-35099942	1.47E-12	-0.336
STOML2	hsa-miR-320a	9:35099984-35099963	1.08E-12	-0.338
PHF19	hsa-miR-15b-5p	9:123618758-123618735	9.74E-11	-0.309
PHF19	hsa-miR-15b-5p	9:123618862-123618838	9.74E-11	-0.309
PHF19	hsa-miR-15b-5p	9:123619735-123619714	9.74E-11	-0.309
BMI1	hsa-miR-200b-3p	10:22619869-22619891	1.08E-13	-0.352
BMI1	hsa-miR-200b-3p	10:22619213-22619236	1.08E-13	-0.352
BMI1	hsa-miR-200b-3p	10:22619751-22619778	1.08E-13	-0.352
INPP5F	hsa-miR-155-5p	10:121587732-121587758	2.01E-11	-0.320
INPP5F	hsa-miR-155-5p	10:121588549-121588571	2.71E-11	-0.318
INPP5F	hsa-miR-155-5p	10:121587892-121587923	2.71E-11	-0.318
PPP3CB	hsa-miR-30e-5p	10:75197720-75197699	1.12E-15	-0.378
PPP3CB	hsa-miR-30e-5p	10:75197049-75197029	1.12E-15	-0.378
PPP3CB	hsa-miR-30c-5p	10:75197056-75197029	3.35E-11	-0.316
PPP3CB	hsa-miR-30e-5p	10:75196719-75196683	3.12E-16	-0.384
PPP3CB	hsa-miR-30c-5p	10:75197182-75197159	3.35E-11	-0.316
PPP3CB	hsa-miR-30c-5p	10:75197721-75197699	3.35E-11	-0.316
MMS19	hsa-miR-29c-3p	10:99218137-99218112	7.16E-12	-0.326
MMS19	hsa-miR-29c-3p	10:99218184-99218168	7.16E-12	-0.326
ACTR1A	hsa-miR-377-5p	10:104239268-104239250	3.57E-31	-0.539
ACTR1A	hsa-miR-377-5p	10:104239070-104239050	3.57E-31	-0.539
ACTR1A	hsa-miR-377-5p	10:104239771-104239749	3.57E-31	-0.539
CCS	hsa-miR-1307-3p	11:66373391-66373414	1.05E-12	-0.338
CCS	hsa-miR-1307-3p	11:66373347-66373369	3.37E-12	-0.331
CCS	hsa-miR-1307-3p	11:66373432-66373451	1.05E-12	-0.338
EED	hsa-miR-30e-5p	11:85989583-85989604	1.29E-15	-0.377
EED	hsa-miR-30e-5p	11:85989691-85989711	1.29E-15	-0.377
EED	hsa-miR-30c-5p	11:85989587-85989604	4.90E-11	-0.314
EED	hsa-miR-30c-5p	11:85989685-85989711	4.90E-11	-0.314
SIDT2	hsa-miR-15a-5p	11:117067012-117067031	2.22E-10	-0.303
CLPB	hsa-miR-200b-3p	11:72004410-72004400	3.13E-10	-0.301
PCSK7	hsa-miR-652-3p	11:117076709-117076684	3.22E-11	-0.316

PCSK7	hsa-miR-652-3p	11:117076548-117076527	3.22E-11	-0.316
PCSK7	hsa-miR-652-3p	11:117076051-117076033	3.22E-11	-0.316
PCSK7	hsa-miR-652-3p	11:117076482-117076462	3.22E-11	-0.316
BACE1	hsa-miR-107	11:117158505-117158480	2.96E-10	-0.301
BACE1	hsa-miR-107	11:117158958-117158933	2.96E-10	-0.301
BACE1	hsa-miR-107	11:117158123-117158096	2.96E-10	-0.301
BACE1	hsa-miR-17-5p	11:117157278-117157255	3.13E-12	-0.332
BACE1	hsa-miR-17-5p	11:117160243-117160223	2.67E-12	-0.333
BACE1	hsa-miR-17-5p	11:117158511-117158487	4.96E-12	-0.329
H2AFX	hsa-miR-320a	11:118965119-118965096	7.67E-13	-0.340
H2AFX	hsa-miR-320a	11:118965012-118964991	7.67E-13	-0.340
RPUSD4	hsa-miR-30d-5p	11:126073232-126073211	1.81E-11	-0.320
RPUSD4	hsa-miR-30d-5p	11:126072971-126072947	1.81E-11	-0.320
RPUSD4	hsa-miR-30d-5p	11:126072488-126072470	1.81E-11	-0.320
SRPR	hsa-miR-30e-5p	11:126133280-126133260	1.39E-13	-0.350
SRPR	hsa-miR-30e-5p	11:126133763-126133737	5.47E-14	-0.356
SRPR	hsa-miR-30e-5p	11:126132900-126132879	1.39E-13	-0.350
NCAPD3	hsa-miR-1-3p	11:134022496-134022469	6.70E-21	-0.436
NCAPD3	hsa-miR-1-3p	11:134022603-134022581	6.70E-21	-0.436
NCAPD3	hsa-miR-1-3p	11:134022763-134022742	6.70E-21	-0.436
ERC1	hsa-miR-1307-3p	12:1599401-1599422	2.52E-10	-0.302
ERC1	hsa-miR-1307-3p	12:1599401-1599422	2.52E-10	-0.302
SSPN	hsa-miR-378a-5p	12:26384585-26384605	6.98E-12	-0.326
SSPN	hsa-miR-378a-5p	12:26384447-26384472	6.98E-12	-0.326
LMBR1L	hsa-miR-30e-5p	12:49491211-49491193	1.26E-12	-0.337
LMBR1L	hsa-miR-30e-5p	12:49491163-49491142	1.26E-12	-0.337
LMBR1L	hsa-miR-30d-5p	12:49491211-49491193	3.45E-11	-0.316
LMBR1L	hsa-miR-30e-5p	12:49490954-49490935	2.28E-16	-0.386
LMBR1L	hsa-miR-30d-5p	12:49491163-49491142	3.45E-11	-0.316
LMBR1L	hsa-miR-30c-5p	12:49490948-49490926	3.00E-15	-0.372
SART3	hsa-let-7g-5p	12:108917069-108917051	3.04E-12	-0.332
RHOF	hsa-miR-148b-3p	12:122217352-122217329	4.53E-11	-0.314
RHOF	hsa-miR-148b-3p	12:122217318-122217297	2.68E-10	-0.302
RHOF	hsa-miR-148b-3p	12:122216582-122216562	2.68E-10	-0.302
KL	hsa-miR-335-5p	13:33639074-33639098	8.79E-15	-0.367
KL	hsa-miR-335-5p	13:33639730-33639757	8.79E-15	-0.367
KL	hsa-miR-335-5p	13:33639412-33639432	8.79E-15	-0.367
RNASEH2B	hsa-miR-940	13:51544336-51544356	3.22E-10	-0.302
RNASEH2B	hsa-miR-940	13:51544118-51544142	3.22E-10	-0.302
RNASEH2B	hsa-miR-940	13:51544274-51544294	3.22E-10	-0.302
IRF9	hsa-miR-106b-5p	14:24635421-24635440	1.34E-13	-0.351
IRF9	hsa-miR-106b-5p	14:24635696-24635717	1.34E-13	-0.351
IRF9	hsa-miR-106b-5p	14:24635480-24635498	1.06E-14	-0.365
EIF2S1	hsa-miR-222-3p	14:67851592-67851612	1.64E-14	-0.363
EIF2S1	hsa-miR-222-3p	14:67852083-67852106	1.64E-14	-0.363
EIF2S1	hsa-miR-21-5p	14:67850630-67850650	2.06E-15	-0.374
EIF2S1	hsa-miR-21-5p	14:67850938-67850959	2.38E-12	-0.333

EIF2S1	hsa-miR-21-5p	14:67852323-67852344	2.38E-12	-0.333
EIF2S1	hsa-miR-222-3p	14:67851242-67851262	1.64E-14	-0.363
DNAL1	hsa-miR-766-3p	14:74163864-74163883	7.37E-11	-0.311
DNAL1	hsa-miR-766-3p	14:74166030-74166061	8.51E-11	-0.310
DNAL1	hsa-miR-766-3p	14:74168871-74168896	8.51E-11	-0.310
ZFYVE21	hsa-miR-92a-3p	14:104199455-104199474	9.39E-12	-0.324
ZFYVE21	hsa-miR-92a-3p	14:104199369-104199390	9.39E-12	-0.324
ZFYVE21	hsa-miR-32-5p	14:104199562-104199583	8.01E-15	-0.367
ZFYVE21	hsa-miR-20a-5p	14:104199779-104199802	3.79E-14	-0.358
ZFYVE21	hsa-miR-20a-5p	14:104199942-104199964	3.79E-14	-0.358
ZFYVE21	hsa-miR-92a-3p	14:104199501-104199522	9.39E-12	-0.324
ZFYVE21	hsa-miR-106b-5p	14:104199903-104199921	4.37E-26	-0.484
ZFYVE21	hsa-miR-17-5p	14:104199901-104199921	1.22E-16	-0.389
ZFYVE21	hsa-miR-93-5p	14:104199942-104199964	1.57E-17	-0.400
ZFYVE21	hsa-miR-17-5p	14:104199942-104199964	1.22E-16	-0.389
ZFYVE21	hsa-miR-17-5p	14:104199779-104199802	1.22E-16	-0.389
ZFYVE21	hsa-miR-25-3p	14:104199502-104199522	6.21E-12	-0.327
ZFYVE21	hsa-miR-20a-5p	14:104199901-104199921	3.79E-14	-0.358
ZFYVE21	hsa-miR-106b-5p	14:104199781-104199802	4.37E-26	-0.484
ZFYVE21	hsa-miR-32-5p	14:104199737-104199763	8.01E-15	-0.367
ZFYVE21	hsa-miR-25-3p	14:104199455-104199474	6.21E-12	-0.327
ZFYVE21	hsa-miR-93-5p	14:104199899-104199921	1.57E-17	-0.400
ZFYVE21	hsa-miR-32-5p	14:104199502-104199522	8.01E-15	-0.367
ZFYVE21	hsa-miR-93-5p	14:104199782-104199802	1.57E-17	-0.400
ZFYVE21	hsa-miR-106b-5p	14:104199944-104199964	4.37E-26	-0.484
ZFYVE21	hsa-miR-25-3p	14:104199369-104199390	6.21E-12	-0.327
ZFYVE26	hsa-miR-93-5p	14:68215150-68215124	6.99E-13	-0.341
ZFYVE26	hsa-miR-93-5p	14:68214720-68214699	6.51E-13	-0.341
ZFYVE26	hsa-miR-106b-5p	14:68214721-68214699	1.99E-10	-0.304
ZFYVE26	hsa-miR-93-5p	14:68214813-68214793	6.99E-13	-0.341
ZFYVE26	hsa-miR-106b-5p	14:68215148-68215124	1.91E-10	-0.304
ZFYVE26	hsa-miR-106b-5p	14:68214817-68214793	1.91E-10	-0.304
ZFYVE26	hsa-miR-93-5p	14:68213513-68213487	6.51E-13	-0.341
ZFYVE26	hsa-miR-106b-5p	14:68213511-68213487	1.99E-10	-0.304
ACTN1	hsa-miR-1-3p	14:69341191-69341171	2.45E-12	-0.333
ACTN1	hsa-miR-1-3p	14:69341247-69341226	2.45E-12	-0.333
ACTN1	hsa-miR-1-3p	14:69340901-69340879	3.73E-34	-0.547
ARIH1	hsa-let-7g-5p	15:72875635-72875656	1.36E-12	-0.337
ARIH1	hsa-let-7g-5p	15:72877475-72877495	1.36E-12	-0.337
ARIH1	hsa-let-7g-5p	15:72878038-72878063	1.36E-12	-0.337
ARIH1	hsa-let-7g-5p	15:72877381-72877401	1.36E-12	-0.337
CD276	hsa-miR-29c-3p	15:74006622-74006643	3.16E-10	-0.301
CD276	hsa-miR-29c-3p	15:74006213-74006230	3.16E-10	-0.301
UNC45A	hsa-miR-125a-5p	15:91497135-91497160	3.41E-10	-0.300
UNC45A	hsa-miR-125a-5p	15:91497266-91497292	3.41E-10	-0.300
UNC45A	hsa-miR-125a-5p	15:91496956-91496977	3.41E-10	-0.300
PKM	hsa-miR-30c-5p	15:72491598-72491575	3.33E-12	-0.331

PKM	hsa-miR-30c-5p	15:72491847-72491825	3.33E-12	-0.331
PKM	hsa-miR-30c-5p	15:72491530-72491508	3.33E-12	-0.331
RAB40C	hsa-let-7g-5p	16:678385-678404	1.35E-12	-0.337
RAB40C	hsa-let-7g-5p	16:678786-678808	1.35E-12	-0.337
RAB40C	hsa-miR-29c-3p	16:677984-678001	9.30E-13	-0.339
RAB40C	hsa-miR-29c-3p	16:678776-678796	9.30E-13	-0.339
RAB40C	hsa-miR-29c-3p	16:677817-677838	9.30E-13	-0.339
RAB40C	hsa-let-7g-5p	16:677713-677734	1.35E-12	-0.337
POLR3E	hsa-miR-98-5p	16:22345157-22345183	4.92E-11	-0.314
POLR3E	hsa-miR-30c-5p	16:22345225-22345252	1.34E-10	-0.307
POLR3E	hsa-miR-98-5p	16:22345114-22345136	4.92E-11	-0.314
POLR3E	hsa-miR-30e-5p	16:22345155-22345179	2.92E-19	-0.419
POLR3E	hsa-miR-30e-5p	16:22345182-22345206	2.92E-19	-0.419
POLR3E	hsa-miR-30e-5p	16:22345229-22345252	2.92E-19	-0.419
PHKB	hsa-miR-15a-5p	16:47733538-47733560	5.33E-11	-0.313
CHD9	hsa-miR-342-3p	16:53359621-53359641	5.02E-11	-0.313
CHD9	hsa-miR-342-3p	16:53359197-53359218	5.02E-11	-0.313
CRISPLD2	hsa-miR-106b-5p	16:84940250-84940270	4.99E-16	-0.382
CRISPLD2	hsa-miR-106b-5p	16:84941101-84941124	2.15E-15	-0.374
CRISPLD2	hsa-miR-676-3p	16:84940255-84940274	1.90E-14	-0.421
CRISPLD2	hsa-miR-106b-5p	16:84940361-84940382	4.99E-16	-0.382
CRISPLD2	hsa-miR-676-3p	16:84942816-84942836	5.95E-14	-0.414
CRISPLD2	hsa-miR-676-3p	16:84941104-84941122	5.95E-14	-0.414
CRISPLD2	hsa-miR-106b-5p	16:84941542-84941565	2.15E-15	-0.374
CRISPLD2	hsa-miR-676-3p	16:84942415-84942434	5.95E-14	-0.414
CRISPLD2	hsa-miR-93-5p	16:84941543-84941565	1.16E-12	-0.338
CRISPLD2	hsa-miR-93-5p	16:84941594-84941621	1.16E-12	-0.338
CRISPLD2	hsa-miR-93-5p	16:84941099-84941124	1.16E-12	-0.338
ZC3H18	hsa-miR-29b-5p	16:88698311-88698329	6.24E-13	-0.344
ZNF200	hsa-miR-30e-5p	16:3273411-3273386	4.05E-13	-0.344
ZNF200	hsa-miR-30e-5p	16:3273385-3273358	4.05E-13	-0.344
DCTPP1	hsa-miR-1-3p	16:30435230-30435208	5.29E-16	-0.382
DCTPP1	hsa-miR-1-3p	16:30435104-30435083	5.29E-16	-0.382
PHLPP2	hsa-miR-141-3p	16:71682306-71682281	8.80E-11	-0.310
PHLPP2	hsa-miR-141-3p	16:71682492-71682470	8.80E-11	-0.310
PHLPP2	hsa-miR-141-3p	16:71681350-71681326	9.17E-11	-0.309
ANKRD13B	hsa-miR-423-5p	17:27941192-27941214	2.25E-10	-0.303
ANKRD13B	hsa-miR-423-5p	17:27941104-27941128	2.25E-10	-0.303
ANKRD13B	hsa-miR-423-5p	17:27941083-27941105	2.25E-10	-0.303
NPEPPS	hsa-miR-141-5p	17:45699583-45699610	4.77E-12	-0.329
NPEPPS	hsa-miR-141-5p	17:45700517-45700540	4.77E-12	-0.329
NPEPPS	hsa-miR-141-5p	17:45699808-45699829	4.77E-12	-0.329
PCTP	hsa-miR-125b-5p	17:53854424-53854445	3.66E-11	-0.316
PCTP	hsa-miR-125b-5p	17:53854545-53854568	3.66E-11	-0.316
PCTP	hsa-miR-125b-5p	17:53853486-53853507	3.66E-11	-0.316
MAP3K3	hsa-miR-106b-5p	17:61771154-61771172	1.52E-15	-0.376
MAP3K3	hsa-miR-93-5p	17:61771152-61771172	4.08E-11	-0.315

MAP3K3	hsa-miR-96-5p	17:61771141-61771167	3.49E-10	-0.300
ANKFY1	hsa-miR-101-3p	17:4069995-4069972	9.03E-15	-0.366
ANKFY1	hsa-miR-101-3p	17:4070298-4070279	9.03E-15	-0.366
ANKFY1	hsa-miR-101-3p	17:4068972-4068949	1.21E-14	-0.365
CTC1	hsa-miR-146b-3p	17:8130781-8130761	6.56E-12	-0.327
CTC1	hsa-miR-146b-3p	17:8131497-8131485	6.56E-12	-0.327
PIGS	hsa-miR-29c-3p	17:26880538-26880517	4.85E-18	-0.405
PIGS	hsa-miR-29c-3p	17:26880717-26880693	4.85E-18	-0.405
PIGS	hsa-miR-29c-3p	17:26881204-26881183	6.12E-18	-0.404
RAB34	hsa-miR-454-3p	17:27041344-27041322	1.28E-15	-0.377
RAB34	hsa-miR-454-3p	17:27041591-27041571	2.61E-10	-0.302
RAB34	hsa-miR-454-3p	17:27041389-27041367	1.24E-10	-0.308
RAB34	hsa-miR-148b-3p	17:27041346-27041321	9.53E-11	-0.309
RAB34	hsa-miR-301a-3p	17:27041345-27041322	3.87E-11	-0.316
RAB34	hsa-miR-454-3p	17:27041653-27041633	2.61E-10	-0.302
CRLF3	hsa-miR-200c-3p	17:29110592-29110571	4.28E-13	-0.344
CRLF3	hsa-miR-200b-3p	17:29110248-29110228	1.44E-11	-0.322
CRLF3	hsa-miR-200c-3p	17:29110246-29110228	4.28E-13	-0.344
CRLF3	hsa-miR-200b-3p	17:29110595-29110571	1.44E-11	-0.322
CRLF3	hsa-miR-200b-3p	17:29111041-29111020	2.06E-11	-0.319
CRLF3	hsa-miR-200c-3p	17:29110549-29110525	4.28E-13	-0.344
EZH1	hsa-miR-93-5p	17:40854543-40854522	1.86E-10	-0.304
EZH1	hsa-miR-16-5p	17:40853730-40853711	1.87E-11	-0.320
EZH1	hsa-miR-93-5p	17:40852364-40852336	2.72E-10	-0.302
EZH1	hsa-miR-15b-5p	17:40854494-40854477	7.70E-14	-0.354
EZH1	hsa-miR-15b-5p	17:40853730-40853711	1.00E-13	-0.352
EZH1	hsa-miR-16-5p	17:40853614-40853594	1.87E-11	-0.320
EZH1	hsa-miR-16-5p	17:40854497-40854477	1.35E-11	-0.322
EZH1	hsa-miR-15b-5p	17:40853619-40853594	1.00E-13	-0.352
EZH1	hsa-miR-93-5p	17:40853344-40853321	2.72E-10	-0.302
TMEM101	hsa-miR-589-5p	17:42089292-42089270	4.64E-13	-0.343
TMEM101	hsa-miR-20b-3p	17:42088902-42088881	8.13E-11	-0.339
TMEM101	hsa-miR-20b-3p	17:42089106-42089075	2.69E-13	-0.379
TMEM101	hsa-miR-20b-3p	17:42088826-42088796	8.13E-11	-0.339
SLC25A39	hsa-miR-103a-3p	17:42397109-42397088	1.47E-11	-0.322
SLC25A39	hsa-miR-103a-3p	17:42397199-42397177	1.47E-11	-0.322
SLC25A39	hsa-miR-103a-3p	17:42397248-42397225	1.61E-11	-0.321
H3F3B	hsa-miR-27a-3p	17:73772858-73772838	1.26E-10	-0.307
H3F3B	hsa-miR-27a-3p	17:73774300-73774279	1.26E-10	-0.307
H3F3B	hsa-miR-27a-3p	17:73772943-73772923	1.26E-10	-0.307
H3F3B	hsa-miR-223-5p	17:73774670-73774650	7.62E-10	-0.302
PRPSAP1	hsa-miR-16-5p	17:74307435-74307409	1.65E-10	-0.305
PRPSAP1	hsa-miR-16-5p	17:74307122-74307103	1.65E-10	-0.305
PRPSAP1	hsa-miR-16-5p	17:74307394-74307374	1.65E-10	-0.305
KIAA1328	hsa-miR-98-5p	18:34803106-34803128	2.70E-10	-0.302
KIAA1328	hsa-miR-98-5p	18:34804317-34804339	3.32E-10	-0.300
KIAA1328	hsa-miR-98-5p	18:34803241-34803264	2.70E-10	-0.302

COLEC12	hsa-miR-148b-3p	18:319865-319839	4.32E-15	-0.370
COLEC12	hsa-let-7g-5p	18:319816-319797	6.94E-14	-0.355
COLEC12	hsa-miR-148b-3p	18:320043-320022	4.32E-15	-0.370
COLEC12	hsa-miR-148b-3p	18:319959-319938	4.32E-15	-0.370
COLEC12	hsa-let-7g-5p	18:319562-319540	6.94E-14	-0.355
COLEC12	hsa-miR-148b-3p	18:319842-319816	4.32E-15	-0.370
COLEC12	hsa-let-7g-5p	18:319443-319419	6.94E-14	-0.355
ANKRD29	hsa-miR-93-5p	18:21180805-21180779	5.00E-11	-0.313
ANKRD29	hsa-miR-93-5p	18:21180109-21180087	4.75E-11	-0.314
ANKRD29	hsa-miR-93-5p	18:21180217-21180197	4.75E-11	-0.314
LRP3	hsa-miR-93-5p	19:33698498-33698519	2.44E-10	-0.303
HNRNPUL1	hsa-miR-191-5p	19:41812678-41812699	1.35E-11	-0.322
HNRNPUL1	hsa-miR-191-5p	19:41812474-41812496	1.35E-11	-0.322
ZNF813	hsa-miR-891a-5p	19:53997144-53997171	4.29E-08	-0.303
ZNF813	hsa-miR-891a-5p	19:53995463-53995482	4.29E-08	-0.303
ZNF813	hsa-miR-891a-5p	19:53997179-53997201	4.29E-08	-0.303
MKNK2	hsa-miR-21-5p	19:2039506-2039485	1.44E-11	-0.322
MKNK2	hsa-miR-21-5p	19:2039568-2039545	1.44E-11	-0.322
MKNK2	hsa-miR-21-5p	19:2037898-2037876	1.78E-11	-0.320
MYO1F	hsa-miR-532-3p	19:8585780-8585763	1.69E-11	-0.321
MYO1F	hsa-miR-532-3p	19:8585955-8585932	1.69E-11	-0.321
MYO1F	hsa-miR-93-3p	19:8585909-8585887	7.91E-12	-0.326
MYO1F	hsa-miR-93-3p	19:8586171-8586150	7.91E-12	-0.326
MYO1F	hsa-miR-93-3p	19:8586272-8586251	7.91E-12	-0.326
MYO1F	hsa-miR-532-3p	19:8586086-8586063	1.69E-11	-0.321
DMPK	hsa-miR-103a-3p	19:46273745-46273736	2.10E-12	-0.334
DMPK	hsa-miR-15a-5p	19:46273745-46273725	1.47E-11	-0.322
DMPK	hsa-miR-15a-5p	19:46273745-46273732	1.47E-11	-0.322
PYGB	hsa-miR-34a-5p	20:25278267-25278287	7.02E-11	-0.311
PYGB	hsa-miR-34a-5p	20:25277401-25277423	7.02E-11	-0.311
PYGB	hsa-miR-34a-5p	20:25278580-25278603	7.02E-11	-0.311
CTSA	hsa-miR-20a-5p	20:44527268-44527293	4.19E-12	-0.330
CTSA	hsa-miR-20a-5p	20:44527162-44527182	4.19E-12	-0.330
CTSA	hsa-miR-20a-5p	20:44527331-44527355	4.19E-12	-0.330
VAPB	hsa-miR-22-3p	20:57022335-57022355	1.66E-14	-0.363
VAPB	hsa-miR-22-3p	20:57021903-57021928	1.66E-14	-0.363
STX16	hsa-miR-429	20:57254327-57254356	8.78E-11	-0.310
STX16	hsa-miR-200b-3p	20:57251662-57251683	3.48E-10	-0.300
STX16	hsa-miR-429	20:57252145-57252166	8.78E-11	-0.310
STX16	hsa-miR-429	20:57251647-57251683	8.78E-11	-0.310
STX16	hsa-miR-200b-3p	20:57252137-57252166	3.48E-10	-0.300
GNAS	hsa-miR-93-5p	20:57485969-57485998	1.25E-10	-0.307
GNAS	hsa-miR-93-5p	20:57486189-57486212	1.25E-10	-0.307
GNAS	hsa-miR-93-5p	20:57485941-57485960	1.25E-10	-0.307
ARFGAP1	hsa-miR-30c-5p	20:61920938-61920959	4.02E-12	-0.330
ARFGAP1	hsa-miR-30c-5p	20:61919254-61919275	2.01E-12	-0.334
ARFGAP1	hsa-miR-30c-5p	20:61920652-61920673	2.19E-12	-0.334

GSS	hsa-miR-125a-5p	20:33516433-33516413	8.72E-11	-0.310
GSS	hsa-miR-125a-5p	20:33516347-33516320	8.72E-11	-0.310
USP25	hsa-miR-200c-3p	21:17251055-17251077	7.52E-13	-0.340
USP16	hsa-miR-320a	21:30426520-30426551	1.09E-12	-0.338
BACE2	hsa-miR-125a-5p	21:42647785-42647810	9.46E-12	-0.324
BACE2	hsa-miR-125a-5p	21:42647995-42648018	9.46E-12	-0.324
BACE2	hsa-miR-125a-5p	21:42648293-42648319	9.46E-12	-0.324
C1QTNF6	hsa-miR-29c-3p	22:37576436-37576415	2.86E-11	-0.317
C1QTNF6	hsa-miR-29c-3p	22:37576641-37576620	2.86E-11	-0.317
C1QTNF6	hsa-miR-29c-3p	22:37576385-37576367	2.86E-11	-0.317
