# Supplementary material for: The effects of alternative splicing on miRNA binding sites in bladder cancer
Source: PLoS One. 2018 Jan 4;13(1):e0190708. doi: 10.1371/journal.pone.0190708 (PMC5754136; doi:10.1371/journal.pone.0190708)
Supplement: S2 Table — (PDF) [file pone.0190708.s005.pdf]

Table S2. Pathway over-representation analysis of 37 genes associated with histology.

Pathway name	*p*-value	FDR *q*-value	Pathway source
Photodynamic therapy-induced HIF-1 survival signaling	8.33E-05	0.00475	Wikipathways
PRC2 complex sets long-term gene silencing through modification of histone tails	0.000435	0.0124	BioCarta
Glucose metabolism	0.00172	0.0291	Reactome
Alcoholism	0.00213	0.0291	KEGG
Amyloid fiber formation	0.00285	0.0291	Reactome
Glucagon signaling pathway	0.00307	0.0291	KEGG
Histone modification	0.00379	0.0297	Wikipathways
PRC2 methylates histones and DNA	0.00417	0.0297	Reactome
Warburg Effect	0.00736	0.0466	Wikipathways
