# Supplementary material for: The effects of alternative splicing on miRNA binding sites in bladder cancer
Source: PLoS One. 2018 Jan 4;13(1):e0190708. doi: 10.1371/journal.pone.0190708 (PMC5754136; doi:10.1371/journal.pone.0190708)
Supplement: S3 Table — (PDF) [file pone.0190708.s006.pdf]

Table S3. Pathway over-representation analysis of 16 genes associated with survival.

Pathway name	*p* -value	FDR *q* -value	Pathway source
Glucose metabolism	1.36E-04	0.00203	Reactome
Photodynamic therapy-induced HIF-1 survival signaling	0.000646	0.00484	Wikipathways
Systemic lupus erythematosus	0.00185	0.00923	KEGG
HIF-1 alpha transcription factor network	0.0026	0.00925	PID
Metabolism of carbohydrates	0.00308	0.00925	Reactome
Alcoholism	0.00558	0.0139	KEGG
Glucagon signaling pathway	0.0068	0.0146	KEGG
Factors involved in megakaryocyte development and plate	0.00791	0.0148	Reactome
